# Systematic analysis of the *Candida albicans* kinome reveals environmentally contingent protein kinase-mediated regulation of filamentation and biofilm formation *in vitro* and *in vivo*

**DOI:** 10.1128/mbio.01249-24

**Published:** 2024-07-01

**Authors:** Juraj Kramara, Min-Ju Kim, Tomye L. Ollinger, Laura C. Ristow, Rohan S. Wakade, Robert Zarnowski, Melanie Wellington, David R. Andes, Aaron G. Mitchell, Damian J. Krysan

**Affiliations:** 1 Department of Pediatrics, Carver College of Medicine, University of Iowa, Iowa City, Iowa, USA; 2 Department of Microbiology, University of Georgia, Athens, Georgia, USA; 3 Department of Medicine, Section of Infectious Disease, University of Wisconsin, Madison, Wisconsin, USA; 4 Department of Medical Microbiology and Immunology, University of Wisconsin, Madison, Wisconsin, USA; 5 Department of Molecular Physiology and Biophysics, Carver College of Medicine, University of Iowa, Iowa City, Iowa, USA; Tel Aviv University, Tel Aviv, Israel

**Keywords:** *Candida albicans*, protein kinase, filamentation, biofilm

## Abstract

Protein kinases are critical regulatory proteins in both prokaryotes and eukaryotes. Accordingly, protein kinases represent a common drug target for a wide range of human diseases. Therefore, understanding protein kinase function in human pathogens such as the fungus *Candida albicans* is likely to extend our knowledge of its pathobiology and identify new potential therapies. To facilitate the study of *C. albicans* protein kinases, we constructed a library of 99 non-essential protein kinase homozygous deletion mutants marked with barcodes in the widely used SN genetic background. Here, we describe the construction of this library and the characterization of the competitive fitness of the protein kinase mutants under 11 different growth and stress conditions. We also screened the library for protein kinase mutants with altered filamentation and biofilm formation, two critical virulence traits of *C. albicans*. An extensive network of protein kinases governs these virulence traits in a manner highly dependent on the specific environmental conditions. Studies on specific protein kinases revealed that (i) the cell wall integrity MAPK pathway plays a condition-dependent role in filament initiation and elongation; (ii) the hyper-osmolar glycerol MAPK pathway is required for both filamentation and biofilm formation, particularly in the setting of *in vivo* catheter infection; and (iii) Sok1 is dispensable for filamentation in hypoxic environments at the basal level of a biofilm but is required for filamentation in normoxia. In addition to providing a new genetic resource for the community, these observations emphasize the environmentally contingent function of *C. albicans* protein kinases.

**IMPORTANCE:**

*Candida albicans* is one of the most common causes of fungal disease in humans for which new therapies are needed. Protein kinases are key regulatory proteins and are increasingly targeted by drugs for the treatment of a wide range of diseases. Understanding protein kinase function in *C. albicans* pathogenesis may facilitate the development of new antifungal drugs. Here, we describe a new library of 99 protein kinase deletion mutants to facilitate the study of protein kinases. Furthermore, we show that the function of protein kinases in two virulence-related processes, filamentation and biofilm formation, is dependent on the specific environmental conditions.

## INTRODUCTION

*Candida albicans* is a commensal of human mucosal surfaces and one of the most common causes of fungal infection (1, 2). Accordingly, *C. albicans* is an extensively studied human fungal pathogen from the point of view of fundamental biology, drug resistance, drug discovery, drug mechanism-of-action studies, vaccine development, mechanisms of pathogenesis, host-pathogen interactions, and innate and adaptive immune responses (3). Historically, the broad sweep of the work focused on *C. albicans* is due, at least in part, to its relatively tractable genetics compared to some other fungal pathogens, particularly in the pre-CRISPR-Cas9 era (4). Indeed, *C. albicans* was one of the first human fungal pathogens for which large-scale mutant collections were available (4–6). Since that time, collections of deletion mutants involving defined functional classes of proteins or near genome-wide coverage of proteins have been developed for *C. glabrata* (7), *Cryptococcus neoformans* (8, 9), and *Aspergillus fumigatus* (10) using a variety of genetic technologies. In *C. albicans*, large-scale collections of strains containing alleles under the control of a tetracycline-regulated promoter (11) or comprised of bar-coded heterozygous deletion mutans (12) are also available. These genetic tools have been invaluable for systematic gene discovery and functional analysis.

One of the most widely employed *C. albicans* mutant collections is the bar-coded transcription factor (TF) deletion set constructed by the Johnson Laboratory (5). It has been used for many single-mutant analyses as well as pooled-competition experiments using both *in vitro* and *in vivo* models of *C. albicans* biology and pathogenesis (13–16). Deletion mutants of regulatory proteins such as TFs are powerful tools for the analysis of complex phenotypes, particularly when combined with network and expression analysis (16, 17). A second broad category of information-rich regulatory proteins is protein kinases (PKs). PKs frequently serve as upstream activators of TFs and function within discrete signaling pathways to relay information within the cell. In addition to their value in the molecular analysis of complex biological processes, PKs are among the most widely drugged targets in modern pharmacology. Because the antifungal pharmacopeia is limited to only three classes of drugs for the treatment of life-threatening fungal infections, PKs represent a reasonable set of proteins to target for the development of new antifungal drugs.

Based on the above considerations, we felt that a systematic set of PK deletion mutants would be a useful addition to the current palate of *C. albicans* genetic resources. Prior to the initiation of this project, Blankenship et al. had reported and characterized a large set of PK transposon insertion mutants (18). While the project was underway, another group reported the use of a PK deletion set (86 PKs) generated in the SC5314 background to identify PKs involved in iron utilization (19). In addition, Lee et al. reported the use of a set of tetracycline-regulated PKs to characterize the kinome relating to *in vitro* filamentation (11). We adopted an approach modeled on that used by Homann et al. to generate the TF deletion set mentioned above (5). Specifically, the PKs were generated in the same genetic background (SN) to allow a more direct comparison of TF and PK phenotypes. We also used the same set of barcode sequences to mark the PK deletion mutants to facilitate the combined use of these libraries in the analysis of a given phenotype without having to re-optimize primer sequences. Finally, the library will be made publicly available through the Fungal Genetics Stock Center.

Here, we report the construction of a non-essential PK deletion set and its characterization under a variety of growth conditions. We have also screened the library to identify PKs required for two related *C. albicans* virulence traits: (i) filamentation and (ii) biofilm formation. We find that each of these traits is dependent upon an extensive network of PKs and identify multiple PKs not previously associated with filamentation or biofilm formation. We also show that two MAPK pathways, the cell wall integrity (CWI) and hyper-osomotic-glycerol (HOG) pathways, play previously underappreciated or unrecognized roles in filamentation with the Mkc1 MAPK of CWI pathway required for the maintenance phase of filamentation *in vitro*. Finally, we demonstrate that the HOG is much more important during *in vivo* biofilm formation than would be predicted from its effect on *in vitro* biofilm formation.

## RESULTS

### Construction of protein kinase deletion set in the SN genetic background

**Fig 1:**
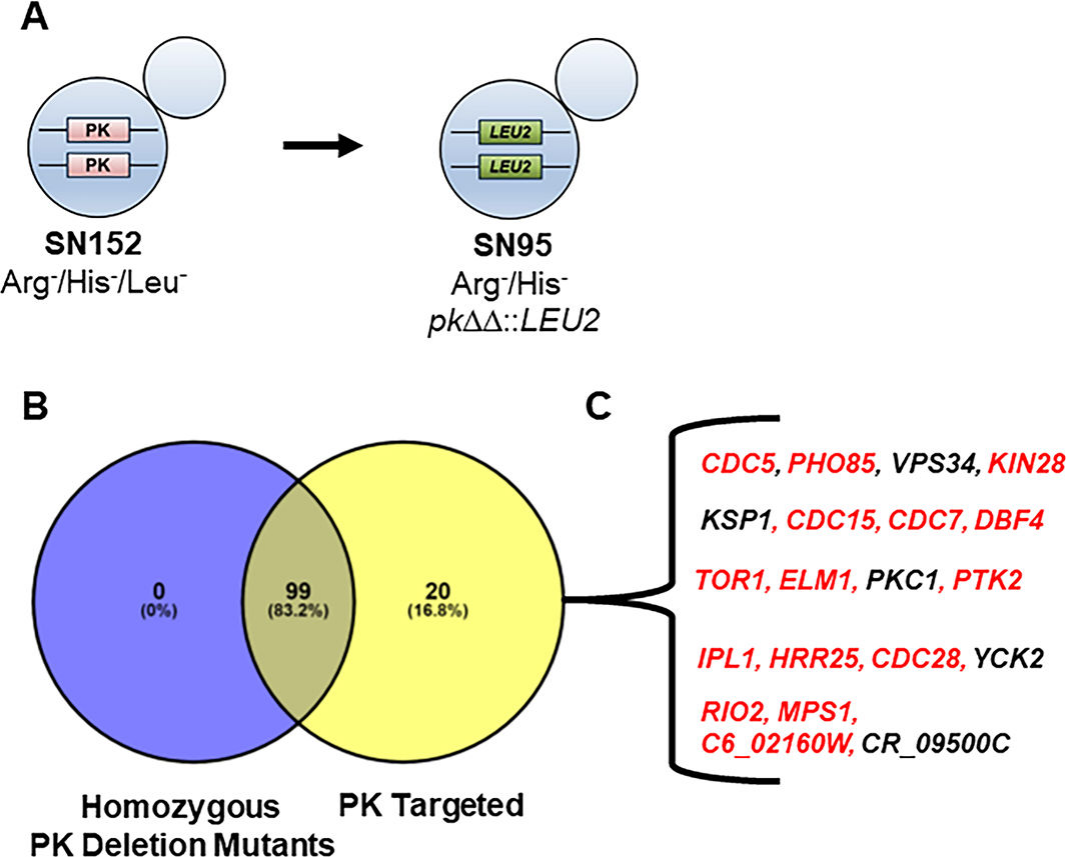
Construction of protein kinase homozygous deletion collection in *C. albicans*. (**A**) Genotypes of parental and homozygous protein kinase deletion strain. (**B**) Venn diagram showing protein kinases targeted (yellow) and homozygous protein kinase mutants constructed (blue). (**C**) The 20 protein kinases for which only heterozygous deletion mutants were obtained. Red font indicates a protein kinase with previously reported to be potentially essential. Black font indicates a protein kinase for which a homozygous deletion mutant has been reported in the literature.

A set of 119 PKs and PK-related genes were collated from databases and previous publications. The parental strain SN152 (Arg^−^, His^−^, Leu^−^) was used to generate the deletion mutants through a CRISPR-Cas9 approach (Fig. 1A). The ORFs were replaced using a *LEU2* auxotrophic marker containing 1 of 30 barcode sequences in the 5′ flanking region. The barcode sequences in the PK mutants are those previously used by Homann et al. in the construction of the TF deletion library (5). Therefore, a common set of barcodes can be used to interrogate both TF and PK deletion sets in the same genetic background. A p5 Illumina adaptor sequence was also placed upstream of the barcodes so that the region could be amplified using a forward primer complementary to the adaptor and a reverse primer homologous to a region of the *LEU2* marker (see Fig. S1A for the schematic of the full construct). The homozygous PK deletion strains match the auxotrophic markers of the SN95 reference strain (Arg^−^, His^−^). Previous studies by Noble and Johnson have shown that the virulence properties of SN95 are comparable to a prototrophic strain in a mouse model of disseminated candidiasis (20).

The open reading frame for the targeted PKs was replaced by a *LEU2*-marked deletion construct using a split-marker approach that results in a pair of inverted repeats on the 5′ and 3′ ends of the *LEU2* gene. The inverted repeats can, therefore, be used to recycle the *LEU2* marker using the CRISPR-Cas9-induced-excision (CRIME) approach developed by Huang and Mitchell while leaving the barcode in place (21). As a result, the PK mutants have a wide variety of genetic markers available for additional strain construction and modification. In addition, they can be readily returned to prototrophy using plasmids available in the community. The deletion set will be available from the Fungal Genetic Stock Center following publication.

Of the 119 ORFs selected, homozygous deletion mutants were obtained for 99 PK genes (Fig. 1B); we obtained heterozygous but not homozygous deletions for 20 of the targeted PKs. Table S1 contains lists all PK homozygous mutants along with the barcode for each mutant as well as the oligonucleotides and guide RNAs used to construct the strain. Because our goal was to construct a large deletion set, we did not exhaustively optimize transformation conditions for PK genes that did not yield homozygous mutants after 2–3 independent attempts. In other organisms, many PKs are essential and, therefore, we compared our set of 20 genes to the literature of essential genes and the specific locus through the Candida Genome Database. As shown in Fig. 1C and Table S2, 15 of the 20 mutants were classified as essential in the literature. Three of the five mutants that were not classified as essential [*PKC1* (22), *YCK2* (23), and *VPS34,* (24)] have been constructed. It is not clear why we could not obtain mutants for these PKs. The literature strains were obtained through sequential deletion of the alleles, whereas we used on one-step deletion approach. It is possible that the heterozygous mutant is able to adapt to the reduced function of the PK and, therefore, is better positioned to tolerate the loss of the gene altogether.

### Quantitative phenotyping of PK mutants under diverse growth conditions using competitive fitness assays

**Fig 2:**
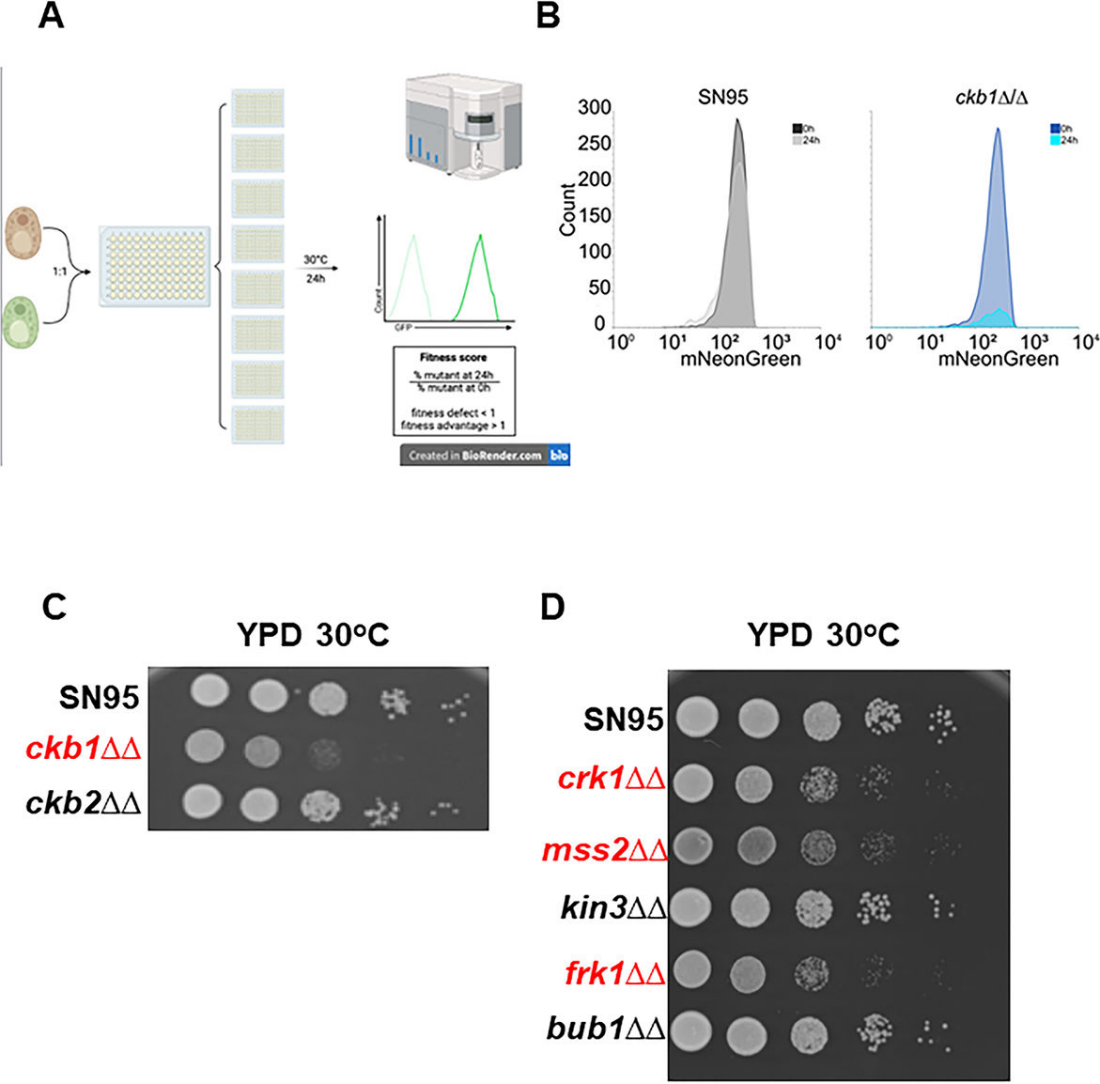
Competitive fitness assay of protein kinase deletion mutants. (**A**) Schematic of flow cytometry-based competitive fitness assay. (**B**) Representative competitive fitness data for *ckb1*∆∆ compared to SN95. (**C**) Spot dilution assay showing phenotype of *ckb1*∆∆ mutant relative to *ckb2*∆∆ and SN95. (**D**) Representative spot dilution growth phenotypes for strains without competitive fitness defects by flow cytometry (black font) and those with reduced fitness (red font).

PKs have a broad range of functions. To begin to characterize these functions, the growth of each PK deletion mutant was compared to the auxotrophic marker-matched SN95 strain using a flow cytometry-based competitive fitness assay (Fig. 2A). SN95 was fluorescently labeled with mNEON using a previously reported plasmid. A 1:1 mixture of mNEON-SN95 and an unlabeled PK mutant was then incubated in microtiter plates under each condition. The ratio of SN95 to PK mutant in the culture at time 0 and 24 hours was determined by gating on the mNEON signal (SN95) and forward scatter (total cells). The PK mutants were first compared to SN95 in rich medium (YPD) at 30°C to assess general growth effects. A representative example of raw data for the *ckb1*∆∆ mutant at 30°C in YPD is shown in Fig. 2B. Seven PK mutants (*ckb1*∆∆, *frk1*∆∆, *crk1*∆∆, *rad53*∆∆, *sok1*∆∆, *cla4*∆∆, and *mss2*∆∆) were less fit than SN95 under these conditions (log_2_ fitness score [FS] ≤ −1, *P* value < 0.05, Student’s *t*-test; Table S3).

To compare the fitness scores to more typical plate-based growth assays, we first examined the discordant phenotype of the regulatory (*CKB1*) and catalytic subunits (*CKB2*) of the B component of the casein kinase complex (Fig. 2C). Consistent with the competitive fitness data, the *ckb1*∆∆ mutant showed a growth defect at 30°C on solid agar YPD media while the *ckb2*∆∆ mutant did not. Three additional mutants with reduced FSs in YPD along with one mutant with no phenotype (*bub1*∆∆) and one with an increased FS (*kin3*∆∆) were also tested. The mutants with reduced FS and WT fitness showed similar phenotypes while the *kin3*∆∆ mutant did not show evidence of an increased growth rate (Fig. 2D); however, it is not clear that this assay would be the most sensitive for such a phenotype. The *frk1*∆∆ and *crk1*∆∆ mutants had very similar FSs (0.26 and 0.30, respectively; Table S3) and showed similar growth phenotypes on solid agar. Based on these data, it appears that the FS provides a reasonably sensitive and quantitative measure of mutant fitness. FSs under all other conditions were normalized to YPD at 30°C to control for intrinsic growth defects.

**Fig 3:**
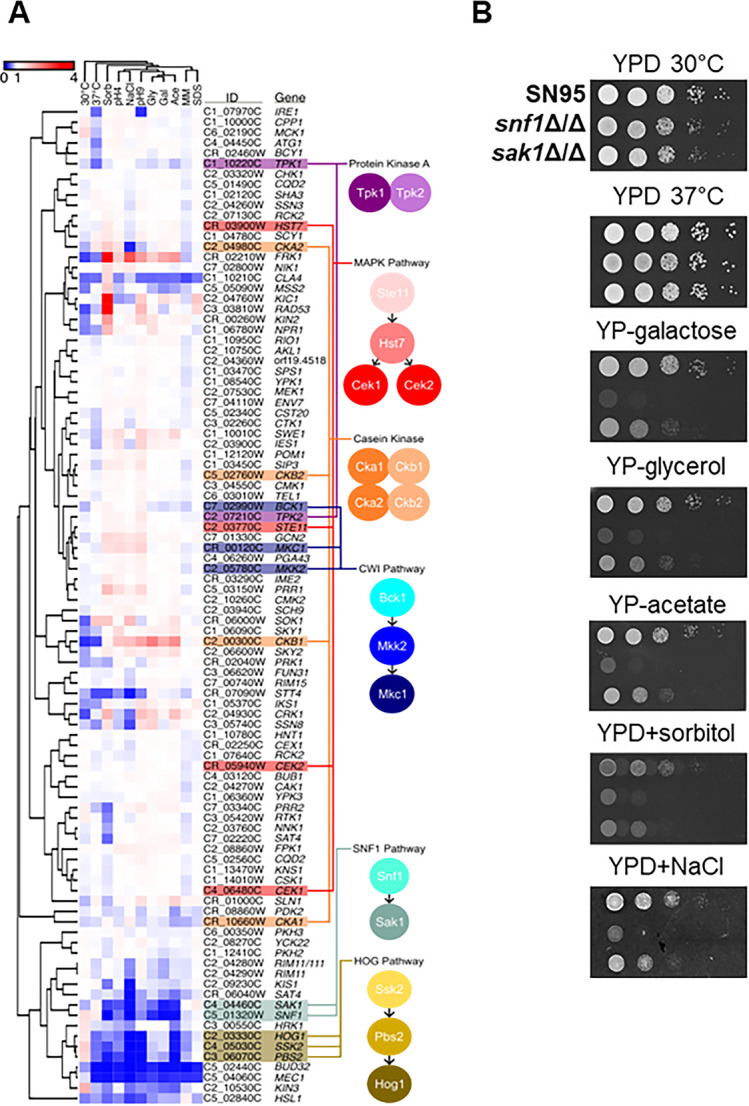
Competitive fitness profile of protein kinase deletion mutants under 10 stress conditions. (**A**) Hierarchical clustering of the protein kinase deletion mutants with the components of specific pathways highlighted. (**B**) Confirmation of Sak1-Snf1 phenotypes on spot dilution assays.

The fitness of each PK mutant was determined in 10 different conditions including temperature, salt, membrane, osmotic, and pH stress. In addition, three non-glucose carbon sources were examined. The results of this screening along with the hierarchical clustering of the mutants are summarized in the heat map shown in Fig. 3A. The components of the cell wall integrity (CWI) and hyperosmotic glycerol (HOG) pathways cluster together as expected (25, 26). Similarly, Sak1 functions upstream of the key metabolic kinase Snf1 and, consistent with those functions, the paired cluster together as do the Sak1-Snf1 pair (27). In addition, two relatively unstudied but paralogous kinases, Sky1 and Sky2 (28), cluster closely as well. By contrast, the two catalytic subunits of the cAMP-PKA kinase (*TPK1* and *TPK2*) and the individual components of the CK2 casein kinase complex (Cka1, Cka2, Ckb1, and Ckb2; Fig. 2C) do not cluster together. The two catalytic subunits of PKA show distinct filamentation phenotypes in *C. albicans* (29). In the case of the latter, the discordance may be because the loss of the regulatory subunits of the CK2 complex leads to distinct phenotypes compared to the loss of function of the catalytic subunits.

Table S4 summarizes the sets of mutants with a log_2_ FS ≤ −1 with a *P* value < 0.05 (Student’s *t*-test) in each of the 10 conditions. Bud32 and Mec1 were the only two mutants with reduced fitness in all conditions. Although neither PK has been extensively studied in *C. albicans*, both Bud32 and Mec1 are conserved across fungi and have been characterized in other systems. Bud32 is part of the kinase, putative endopeptidease, and other proteins of small size (KEOPS) complex that regulates multiple critical cellular functions such as telomere regulation, cell cycle progression, and chromatin/transcription (30). Mechanistically, at least part of the pleomorphoric functions of the KEOPS complex is due to its role in tRNA modification. Although *C. albicans* Bud32 has not been previously studied in detail, the extensive phenotypes observed for the *bud32*∆∆ mutant are consistent with reported functions in other fungi such *S. cerevisiae* and *Cryptococcus neoformans*. Mec1 is part of the Mec1-Rad53 DNA damage signaling pathway and, therefore, the pleomorphic phenotypes are not surprising (31).

Because *C. albicans SNF1* was long thought to be essential, its effect on non-carbon metabolism phenotypes has not been investigated (27). In *S. cerevisiae*, Snf1 is required for cellular responses to other environmental stresses in addition to glucose limitation and is regulated by *SAK1* (32). Consistent with their known functions during growth on non-glucose carbon sources, both the *snf1*∆∆ and *sak1*∆∆ mutant grew poorly on media containing galactose, glycerol, and acetate (Fig. 3B). Both the *sak1*∆∆ and *snf1*∆∆ mutants have reduced fitness under osmotic, alkaline, and acidic stresses (Fig. 3B). However, unlike the *S. cerevisiae snf1*∆ mutant, the *C. albicans snf1*∆∆ mutant is not sensitive to elevated temperature (Fig. 3B). Although loss of Snf1 affects many cellular processes, it does not lead to a global loss of fitness.

Finally, the utility of phenotypic hierarchical clustering has significant limitations, particularly in cases where kinases thought to function together do not cluster as expected. This is most likely due to the relatively small set of conditions we used for our analysis and the broad functions of PKs. PKs that cluster together are likely to have related functions; however, the lack of clustering does not mean that they do not have related functions under other conditions. For example, despite functioning in the same DNA damage pathway, the *mec1*∆∆ and *rad53*∆∆ mutants do not have overlapping phenotypes; however, we did not examine DNA damage conditions and, therefore, this discordance is likely due to differences in compensatory responses or other indirect processes. As such, the clustering results have high specificity in the sense that clustered PKs are likely to have similar functions under those specific conditions, but lacks sensitivity and cannot exclude the likelihood that PKs have related functions in other conditions.

### An extensive network of PKs regulates *C. albicans* hyphal morphogenesis *in vitro*

The ability to transition between yeast and hyphal morphologies is one of the most well-studied *C. albicans* virulence traits. Many genes including PKs have been identified as contributors to this phenotype through gene-specific studies as well as large-scale studies. For example, Lee et al. recently reported a screen of strains containing tetracycline-regulated PK mutants for those that regulate filamentation (11). To systematically characterize the filamentation phenotypes of our PK deletion mutant set, we used a plate-based primary screen in which the PK mutants were spotted on YPD, Spider medium, RPMI, and RPMI + 10% bovine calf serum (BCS) agar plates. The plates were incubated at either 30°C or 37°C with an auxotrophic marker-matched reference strain (SN95) control on each plate; the screen was performed in biological duplicate. Except for the YPD experiments, plates were photographed on incubation days 3, 4, and 5. Each strain was scored from −3 to 0 for peripheral colony filamentation using the system represented in Fig. S1B. Negative scores were based on filamentation at day 5 while mutants were classified as having +1 or +2 if they showed an increased filamentation score prior to day 5. The YPD plates were incubated at either 30°C or 37°C and photographed on days 1, 2, and 3 to identify colonies with evidence of filamentation when SN95 showed none. We recognize that central wrinkling is also a widely used filamentation phenotype but scoring this phenotype was poorly reproducible in our hands while filamentation was consistent between replicates (5, 6). Therefore, we focused on peripheral filamentation.

**Fig 4:**
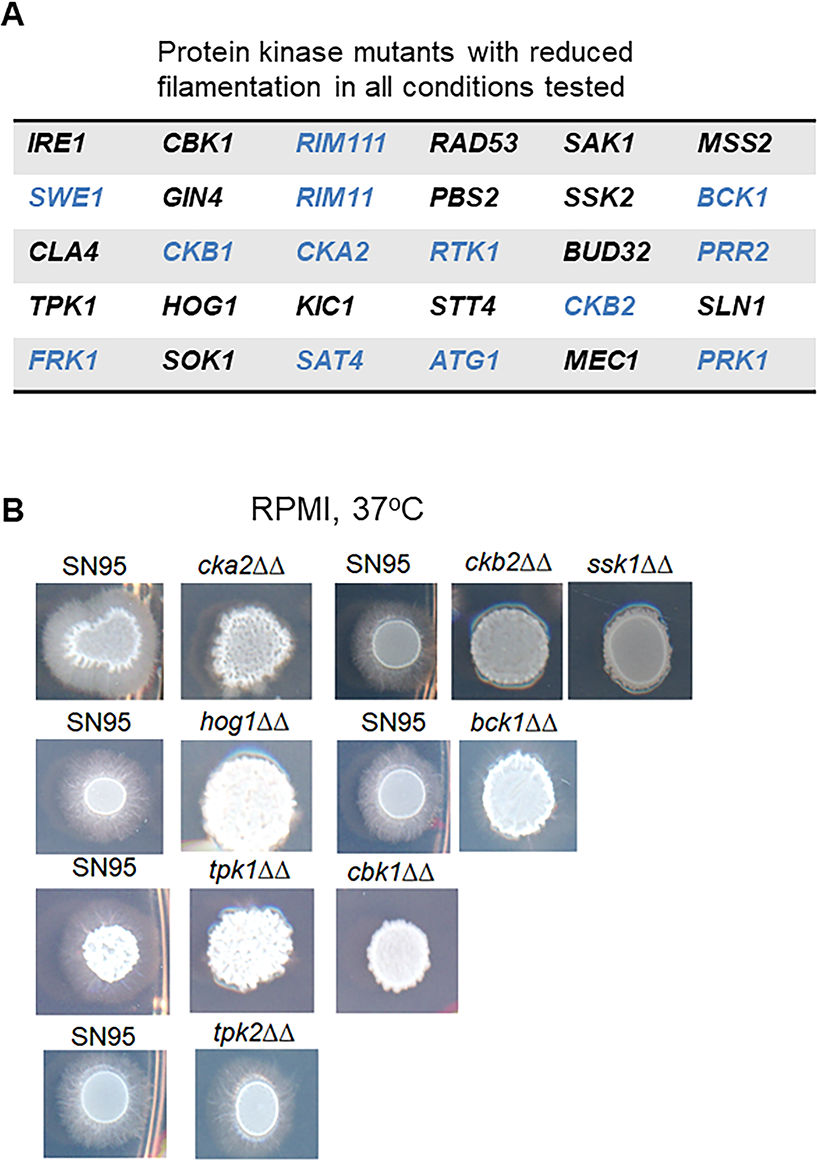
Summary of filamentation phenotypes of protein kinase deletion mutants and representative phenotypes. (**A**) Table of protein kinase mutants with reduced filamentation on all filament-inducing conditions tested. Black font indicates protein kinases previously implicated in filamentation and blue font indicates protein kinases not previously reported to play a role in filamentation. (**B**) Representative phenotypes of the reference strain SN95 and protein kinase deletion mutants on RPMI medium incubated at 37°C.

Remarkably, 88 of 99 PK mutants tested had altered filamentation on at least one of the six filament-inducing conditions (the filamentation phenotypes for each strain are summarized in Table S5). In comparison, 40/155 transcription factor mutants had altered filamentation according to the screen reported by Homann et al. (5). Of this set, 18 mutants showed increased filamentation and 69 had a reduced filamentation score under at least one condition. All of the mutants with growth defects at either 30°C or 37°C had reduced filamentation. However, all mutants with growth defects formed colonies on the solid agar plates so it is not clear how much the temperature sensitivity contributed to the reduced filamentation. Although no PK mutant was hyper-filamentous on all conditions, 30 PK mutants had negative filamentation scores on all six hypha-inducing media and temperature combinations (Fig. 4A). Of the 30 PK mutants that showed reduced filamentation on all six conditions, 12 PK mutants (we excluded *MEC1*, *RAD53*, and *BUD32* due to their pleomorphic growth defects) had not been previously reported to show decreased filamentation through a search of the CGD database and PubMed literature.

In this set of PK mutants, only two mutants showed evidence of filamentation on non-inducing conditions (YPD medium): *cak1*∆∆ and *swe1*∆∆. Both of these mutants showed dramatically increased colony wrinkling that was evident after 24 hours at 37°C but not at 30°C (Fig. S1C). We did not observe peripheral invasion for these strains up to 72-hour incubation. Cak1 has previously been shown to repress filamentation (11, 33) while *swe1*∆∆ has been reported to have no filamentation phenotypes (34). As noted in Fig. 4A, the *swe1*∆∆ mutant has decreased filamentation under inducing conditions and increased filamentation under non-inducing conditions. Swe1 is involved in cell cycle processes and elongated cell morphology is a well-established phenotype of cell cycle mutants, providing a possible explanation for the phenotype.

Representative filamentation phenotypes on RPMI at 37°C are shown in Fig. 4B. The *CKA2* deletion was reported to have no phenotype in another background but the filamentation conditions tested were not reported (35). No previous studies on the filamentation of the *ckb2*∆∆ mutant have been reported. Our data indicate that the function of the CK2 complex extends to filamentation and is likely to contribute to the reduced virulence of CK2 complex mutants (35). The Hyperosmotic Glycerol pathway (HOG) is annotated as suppressing filamentation in the CGD database. However, the literature indicates that the role of *HOG1* in filamentation is more complex. In their initial characterization of the *hog1*∆∆ mutant, the Pla laboratory found that its phenotype was dependent on conditions. On solid Spider medium, the *hog1*∆∆ mutant shows no filamentation while the addition of low concentrations of serum to liquid YPD led to early filamentation of the *hog1*∆∆ mutant relative to its parental strain (36); the latter phenotype has led, in part, to its classification as a repressor of filamentation. We found that three kinases in the HOG pathway (*SSK2*, *PBS2*, and *HOG1*) all showed reduced filamentation with *HOG1* and *SSK1* mutants deficient under all conditions tested (Fig. 4A). The reduced filamentation phenotypes for *hog1*∆∆ and *ssk1*∆∆ mutants are shown in Fig. 4B and, therefore, suggest that the HOG pathway plays an under-appreciated role in the positive regulation of filamentation and a more limited role as a repressor of filamentation.

The literature is also somewhat mixed with respect to the role of the Cell Wall Integrity pathway (CWI) during filamentation. In their initial report on the *mkc1*∆∆ mutant, Navarro-Garcia found that it had reduced filamentation on solid Spider medium (37). Kumamoto demonstrated that Mkc1 plays a key role in contact-induced filamentation (38). By contrast, Xie et al. found that, among the kinases of the CWI pathway (22), the *pkc1*∆∆ mutant but neither the *bck1*∆∆ nor *mkc1*∆∆ had reduced filamentation in YPD + 10% BCS. On solid medium, we found that mutants of each CWI component MAP-family kinase (*BCK1*, *MKK2*, and *MKC1*) had reduced filamentation (Table S5) on at least one condition with the *bck1*∆∆ mutant deficient in each condition (Fig. 4A and B). The fact that the *bck1*∆∆ mutant has consistently reduced filamentation on multiple inducing conditions but mutants of the other CWI pathway components do not suggest that they play roles other than simply regulating the next PK in the cascade.

Lastly, it is also important to note that our screening results recapitulated phenotypes for many of the previously reported PK mutants. For example, the venerable cAMP-protein kinase A pathway mutants matched the previously reported pattern in which the *TPK1* mutant has reduced filamentation under most conditions while the *TPK2* mutant filaments normally (Fig. 4B) (29). The RAM pathway component kinases *KIC1* and *CBK1* (Table S5; Fig. 4B) are also both required for filamentation (39). Overall, the role of PKs in filamentation appears to be highly dependent upon the specific conditions under which *C. albicans* filamentation is induced.

### PK-*NRG1* genetic interactions provide insights into the timing of PK functions during filamentation

**Fig 5:**
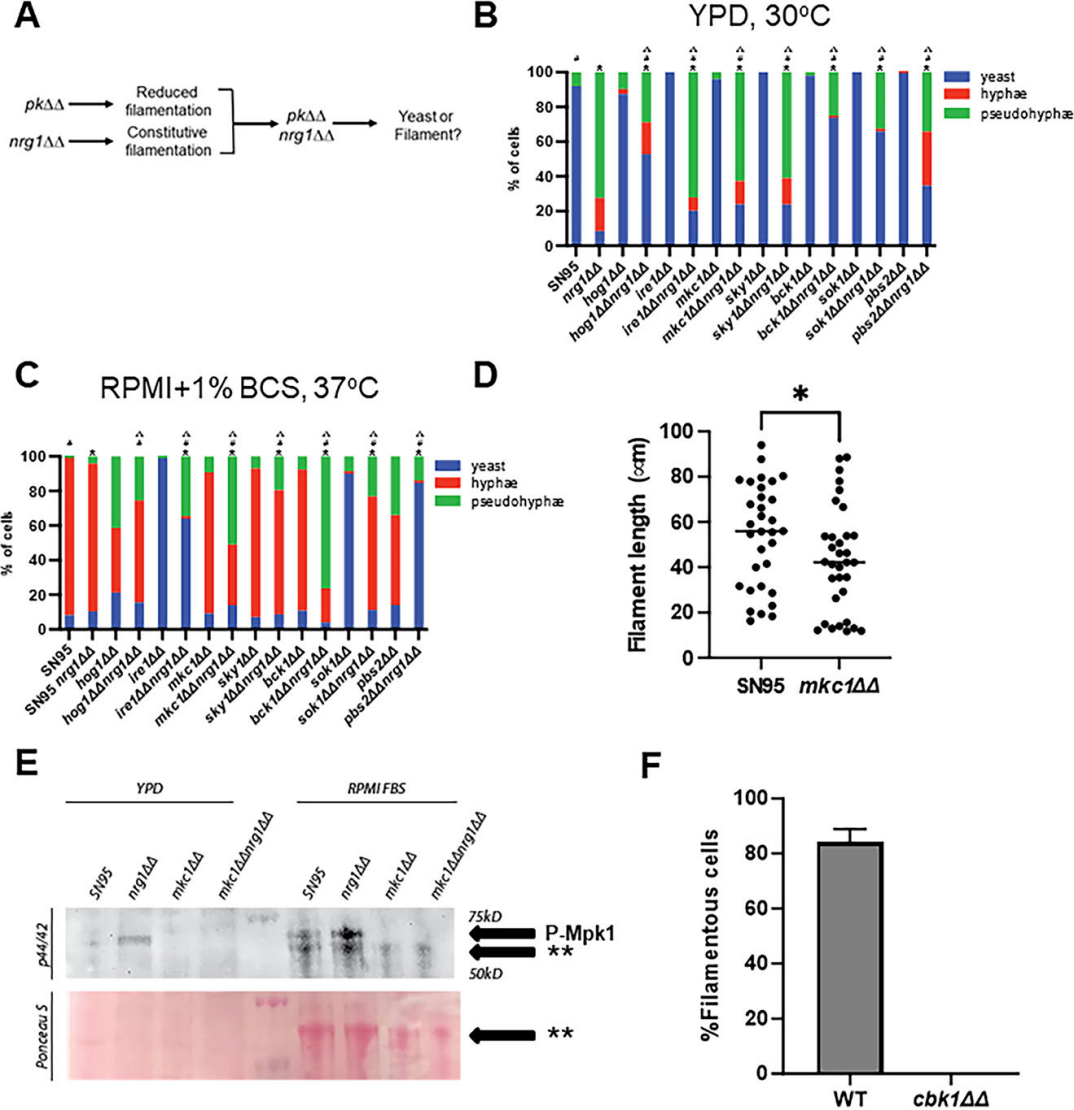
Genetic interactions of protein kinase mutants with *nrg1*∆∆ mutant reveal role for cell wall integrity pathway during hyphal extension. (**A**) Strategy to assess the timing of protein kinase function during filamentation using genetic interactions with *nrg1*∆∆ mutant. (**B and C**) The indicated strains were incubated in YPD at 30°C (**B**) or RMPI + 1% bovine calf serum (**C**) and the distribution of yeast, pseudohyphae, and hyphae as determined by microscopy on fixed samples. The bars indicate the means of two independent experiments with greater than 100 cells counted per experiment. Chi-squared tests were used to evaluate the statistical significance of comparisons (*P* < 0.05): * indicates comparison to protein kinase single deletion mutant; # indicates comparison to *nrg1*∆∆ mutant; ∆ indicates comparison to SN95. (**D**) The length of hyphae formed after 4-hour induction in RPMI + 1% bovine calf serum was determined as described in Materials and Methods. Statistical significance was determined by Mann-Whitney; * indicates *P* < 0.05. (**E**) Western blot analysis of Mkc1 phosphorylation in the indicated strains using anti-p42/44 antibody in the indicated strains after incubation in YPD at 30°C or RMPI + 10% BCS at 37°C for 4 hours. Ponceau staining was used to assess loading. ** indicates a non-specific band observed in serum-induced samples. The blots are representative of three independent experiments. (**F**) The percentage of filamentous cells in WT or *cbk1*∆∆ mutant strains 24 hours after inoculation of mouse ears.

The conserved repressor Nrg1 plays a central role in the initiation of *C. albicans* filamentation (40, 41). Upon transfer to filament-inducing conditions, the Nrg1 protein is rapidly degraded and the expression of its gene, *NRG1*, is reduced. This leads to activation of the hyphal transcriptional program and formation of hyphae (42). Constitutive expression of *NRG1* from a heterologous promoter blocks filament formation *in vitro* and during infection (43). Conversely, deletion of *NRG1* leads to initiation of filamentation (pseudohyphae) in the absence of typical hypha-inducing stimuli (40, 41). Therefore, candidate genes could function either upstream of the relief of Nrg1 repression or downstream: the filamentation phenotypes of mutations in genes functioning upstream should be suppressed by deletion of *NRG1* while those involved in positively regulating the hypha program after relief of Nrg1 repression should have altered filamentation relative to the *nrg1*∆∆ mutant. Therefore, we constructed a set of *pk*∆∆ *nrg1*∆∆ double mutants and characterized their effects on *nrg1*∆∆ mutant pseudohyphae formation in YPD and the effect of the *nrg1*∆∆ mutation on reduced hypha formation of PK mutants during hyphae induction (Fig. 5A). For these experiments, we used liquid culture-based induction conditions to quantitatively characterize the distribution of cellular morphotypes. It is important to note that filamentation and invasion of solid medium, for instance, do not always correlate.

We chose 7 PK mutants with filamentation phenotypes and generated *pk*∆∆ *nrg1*∆∆ double mutants. The HOG (*pbs2*∆∆ and *hog1*∆∆ mutants) and CWI pathway (*mkc1*∆∆ and *bck1*∆∆ mutants) mutants were chosen because their roles have not been extensively studied. Similarly, at the time of our work, neither Ire1 nor Sky1 had been extensively characterized. Sok1 was also included because deletion of *NRG1* has been shown to restore filamentation to the *sok1*∆∆ mutant (44). We first examined the effect of the seven PK mutations on pseudohyphae formation by the *nrg1*∆∆ mutant in YPD at 30°C and compared the results to their corresponding *nrg1*∆∆ double mutant along with SN95 and the *nrg1*∆∆ single mutant.

As expected, the *nrg1*∆∆ mutant formed primarily pseudohyphae along with a few hyphae and very few yeast under these conditions (Fig. 5B). The seven PK mutants grew almost exclusively as yeast with rare pseudohyphae. The *hog1*∆∆ and *pbs2*∆∆ mutants were the only mutants to form quantifiable hyphae to any extent (<10%). These observations may be related to previous reports indicating that HOG mutants form increased hyphae under specific conditions (36). Each PK *nrg1*∆∆ double mutant formed an increased proportion of yeast relative to the parental *nrg1*∆∆ mutant (Fig. 5B). Hog1, Bck1, and Sok1 had the strongest effects on pseudohyphae formation under these conditions. Ire1 and Mkc1, on the other hand, had minimal effects on pseudohyphae formation (Fig. 5B). These data indicate that Hog1, Bck1, and Sok1 modulate pseudohyphae formation after relief of Nrg1 repression under non-inducing conditions. The phenotype of the *sok1*∆∆ *nrg1*∆∆ mutant is quite striking. Under inducing conditions, the Sok1 kinase is thought to phosphorylate Nrg1 leading to its degradation and, consequently, relief of hyphal expression (44). If that were the only role that it plays, then the *sok1*∆∆ *nrg1*∆∆ mutant should phenocopy the *nrg1*∆∆ mutant. The *sok1*∆∆ *nrg1*∆∆ mutant shows a dramatic increase in yeast formation relative to *nrg1*∆∆, indicating Sok1 must have at least some distinct roles during pseudohyphae formation.

Next, we asked whether the deletion of *NRG1* suppressed the filamentation defects of PK mutants. To do so, we exposed the *nrg1*∆∆ double mutants to filament induction in RPMI + 1% BCS instead of 10% BCS to increase sensitivity for more subtle phenotypes. The *sok1*∆∆ *nrg1*∆∆ double mutant forms hyphae to a similar extent as the *nrg1*∆∆ mutant while the *sok1*∆∆ single mutant forms almost no hyphae (Fig. 5C). This is consistent with previous observations and the model that Sok1 mediates degradation of Nrg1 during hyphae initiation (44). Like the *sok1*∆∆ mutant and consistent with plate-based assays, the *ire1*∆∆ mutant forms essentially no hyphae or pseudohyphae under these inducing conditions (Fig. 5C). In this case, however, deletion of *NRG1* does not restore hyphae formation as the *ire1*∆∆ *nrg1*∆∆ double mutant forms predominately yeast and pseudohyphae. Thus, it appears that Ire1 contributes to the initiation of filamentation after relief of Nrg1 repression. It is also clear that Ire1 is critically required for *C. albicans* filamentation but not pseudohyphae formation in the *nrg1*∆∆ mutant (Fig. 5B).

Deletion of the HOG pathway PKs Hog1 and Pbs2 caused increased pseudohyphae relative to hyphae with a very minor increase in yeast (Fig. 5C). In this case, the two components of this linear pathway had very similar phenotypes. The *nrg1*∆∆ double mutants of these PKs, however, had very different phenotypes. In the case of the *hog1*∆∆ mutant, deletion of *NRG1* had a minor effect on the distribution of morphotypes relative to the *hog1*∆∆ single mutant (Fig. 5C). The inability of a *nrg1∆∆* mutation to affect the *hog1*∆∆ mutant phenotype is consistent with Hog1 functioning mainly after relief of Nrg1 repression. In stark and surprising contrast, the *pbs1*∆∆ *nrg1*∆∆ mutant forms almost no hyphae and is predominantly in yeast form. This phenotype is consistent with Pbs2 also functioning downstream of the step relieving Nrg1.

The distinctions between the phenotypes of the *hog1*∆∆ *nrg1*∆∆ and *pbs1*∆∆ *nrg1*∆∆ mutants are striking. First, these observations indicate that Pbs2 must have functions in addition to activating Hog1 during filamentation and is a conclusion that has precedent in other studies of the HOG pathway in *C. albicans*. Second, the *pbs2*∆∆ mutant forms hyphae and, consequently, the strong reduction in filamentation in the *pbs2*∆∆ *nrg1*∆∆ mutant indicates that Pbs2 is required for initiation of filamentation only in the absence of *NRG1*. As such, this is a phenotype unique to the double mutant and, therefore, is a synthetic genetic interaction. The synthetic phenotype between Nrg1 and Pbs2, therefore, implies that Nrg1 must have a role beyond repressing the initiation of filamentation. Nrg1 cannot simply be an on-off switch for filamentation; otherwise, all of the double mutant phenotypes would be explainable by an additive interaction between the phenotypes of the PK mutant and the *nrg1*∆∆ mutants. Supporting this model is the fact that Nrg1 protein is only transiently reduced at the initiation of filamentation but is readily detectable after 60 min to 90 min of hyphal induction in most media (45). Furthermore, the expression of *NRG1* is reduced during filamentation but remains readily detectable throughout filamentation *in vitro* and *in vivo*. Thus, there is only a brief window during which Nrg1 levels are dramatically low and there is an opportunity for Nrg1 to play additional roles beyond its canonical function.

Further supporting the above assertion are the phenotypes of the *nrg1*∆∆ double mutants with CWI pathway PKs Bck1 and Mkc1. Consistent with previously reported data in liquid YPD + 10% BCS hyphal induction conditions (22), neither the *bck1*∆∆ nor the *mkc1*∆∆ mutant had a significant reduction in hyphae formation in RPMI + 10% BCS (Fig. 5C). The *bck1*∆∆ *nrg1*∆∆ and *mkc1*∆∆ *nrg1*∆∆ double mutants, on the other hand, formed significantly fewer hyphae relative to either single mutant with a corresponding increase in pseudohyphae (Fig. 5C). Once again, these are synthetic phenotypes and, as such, imply that the CWI pathway is critically important during the period of the hyphal program when Nrg1 repression is relieved.

### The CWI pathway contributes to the maintenance phase of hyphal morphogenesis *in vitro* but not *in vivo*

To test the hypothesis that the CWI pathway functions primarily during hyphal elongation under liquid inducing conditions, we first measured the length of hyphae formed under these conditions. Supporting the hypothesis, the length of the filaments formed by SN95 is significantly longer than the filaments formed by the corresponding *mkc1*∆∆ mutant after 4 hours of *in vitro* induction (Fig. 5D). If the CWI pathway is important for the maintenance or elongation phase of hyphal morphogenesis then phosphorylation of Mkc1, the terminal kinase, should occur during filamentation. To test this hypothesis, we used an antibody that recognizes the phosphorylated form of Mkc1. After 4 hours of hyphae induction in RPMI +10% BCS, Mkc1 phosphorylation increased relative to yeast phase cells in rich medium (Fig. 5E); this is also consistent with previous data reported by Kumamoto in YPD medium (38). Because deletion of *NRG1* leads to constitutive induction of the filamentation program and deletion of *MKC1* in the *nrg1*∆∆ background increases the proportion of yeast (Fig. 5B), we asked whether Mkc1 was phosphorylated in the *nrg1*∆∆ mutant under non-inducing conditions. Indeed, Mkc1 is phosphorylated in the *nrg1*∆∆ mutant, suggesting it functions after relief of Nrg1 repression (Fig. 5E). These data support the conclusion that the CWI pathway is activated during filamentation and contributes to the maintenance phase of hyphal morphogenesis in the presence of external hypha-inducing stimuli. The synthetic genetic interactions of the HOG and CWI pathway mutants with Nrg1 also suggest that Nrg1 has roles in filamentation beyond repression of the hyphal program.

Lastly, we tested the role of Mkc1 during filamentation *in vivo* using an *in vivo* imaging assay of filamentation in the sub-epithelial stroma of mouse ears (46). The ear tissue is injected with a 1:1 mixture of mNEON labeled reference strain and a mutant strain labeled with iRFP; the ears are then imaged 24 hours post-infection and both % filamentous cells and length of the filaments are determined as described in Materials and Methods. In the *mkc1*∆∆ mutant, the extent and length of filament formation were not statistically different (Fig. S1D and E). Based on time course experiments, the 4-hour *in vitro* and 24-hour *in vivo* time points are points where the ratio of yeast to filaments and the lengths have reached a steady state (47). In contrast to the *mkc1*∆∆, the deletion of *CBK1*, a PK required for filamentation under all *in vitro* conditions tested, formed essentially no filaments *in vivo* (Fig. 5F; Fig. S2F).

### *C. albicans* biofilm formation requires a diverse set of protein kinases

**Fig 6:**
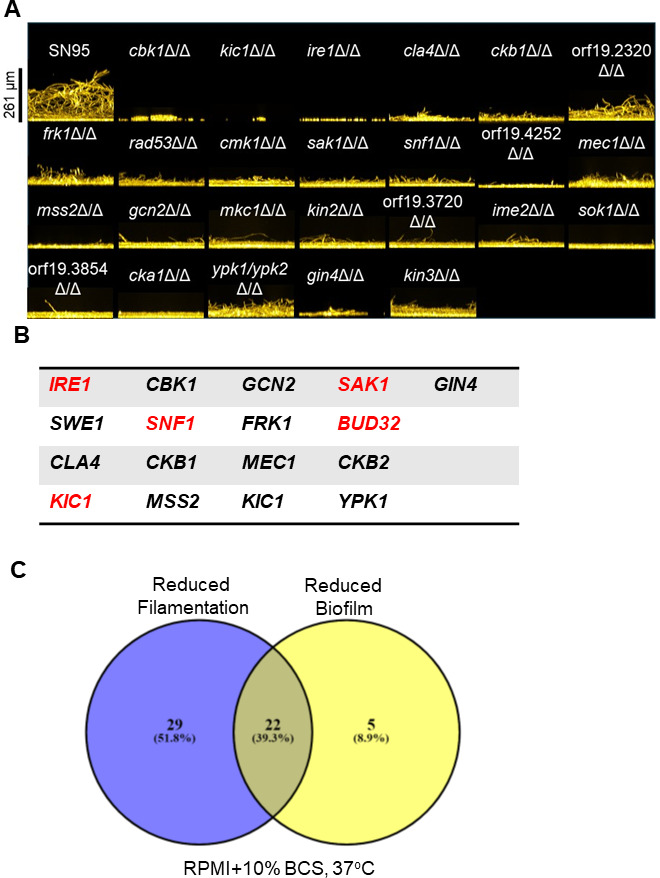
An extensive network of protein kinases governs biofilm formation. (**A**) Representative protein kinase mutants with severe or moderate phenotypes based on the imaging assay used to screen the deletion collection. Biofilms were formed in RPMI + 10% bovine calf serum at 37°C for 24 hours in microtiter plates, stained with Calcofluor white, and imaged as described in Materials and Methods. The cross-section images of the biofilm are shown. (**B**) Protein kinases whose deletion mutants have reduced biofilm formation in both Spider medium and RPMI + 10% bovine calf serum at 37°C. Red font indicates severe phenotype. (**C**) Venn diagram showing the overlap between protein kinase mutants with reduced filamentation and biofilm phenotypes in RPMI + 10% bovine calf serum at 37°C.

*C. albicans* forms biofilms on inanimate surfaces such as intravascular catheters, medical devices and prosthetics, and dental appliances to cause a variety of human diseases (48). Biofilms also form on animate surfaces in the host, contributing to oropharyngeal candidiasis for example (49). To identify the network of PKs required for *in vitro* biofilm formation, we used a moderate throughput imaging assay in two commonly used media: Spider medium and RPMI + 10% BCS. Biofilm formation was assayed after 24-hour incubation at 37°C (50). Mutants were scored according to the thickness of the biofilm visualized by cross-section images of the well bottom based on two replicates per mutant and relative to SN95 strains imaged at the same time. We classified the mutants as having minor (−1), moderate (−2) or severe defects (−3). Figure 6A shows the reference strain along with representative examples of PK mutants with moderate or severe defects in RPMI + 10% BCS. Images for all strains tested in RPMI + 10% BCS are provided in Fig. S2A and B while scoring of strains in the two conditions is summarized in Table S5.

In RPMI + 10% BCS, 30 PK mutants had reduced biofilm formation with five having severe effects (*IRE1*, *KIC1*, *SAK1*, *SNF1*, and *BUD32*) while 22 PK mutants had reduced biofilm formation in Spider medium with two having severe defects (*KIC1* and *BUD32*). Seventeen PK mutants had defects in both media (Fig. 6B). Of this set of mutants, four (*sak1*∆∆, *snf1*∆∆, *bud32*∆∆, and *mec1*∆∆) have growth defects under a variety of conditions (Fig. 3A; Table S3) which likely contribute to their poor biofilm formation. We did not identify PK mutants with increased biofilm density. Because filamentation is part and parcel of *C. albicans* biofilm formation (49), we compared the set of PKs required for filamentation on RPMI + 10% BCS with those required for biofilm formation in the same medium (Fig. 6C). The majority of PK mutants with reduced biofilm formation under these conditions also had reduced filamentation (22/27). However, less than 50% of the PK mutants with reduced filamentation in RPMI + 10% BCS also showed reduced biofilm formation. Interestingly, although only a handful of PK mutants have been tested in a mouse model of oropharyngeal candidiasis, an example of *in vivo* biofilm formation, the *frk1*∆∆ mutant has been shown by Naseem et al. to have a virulence defect in this setting, suggesting it has a general role in biofilm formation in multiple conditions (50).

**Fig 7:**
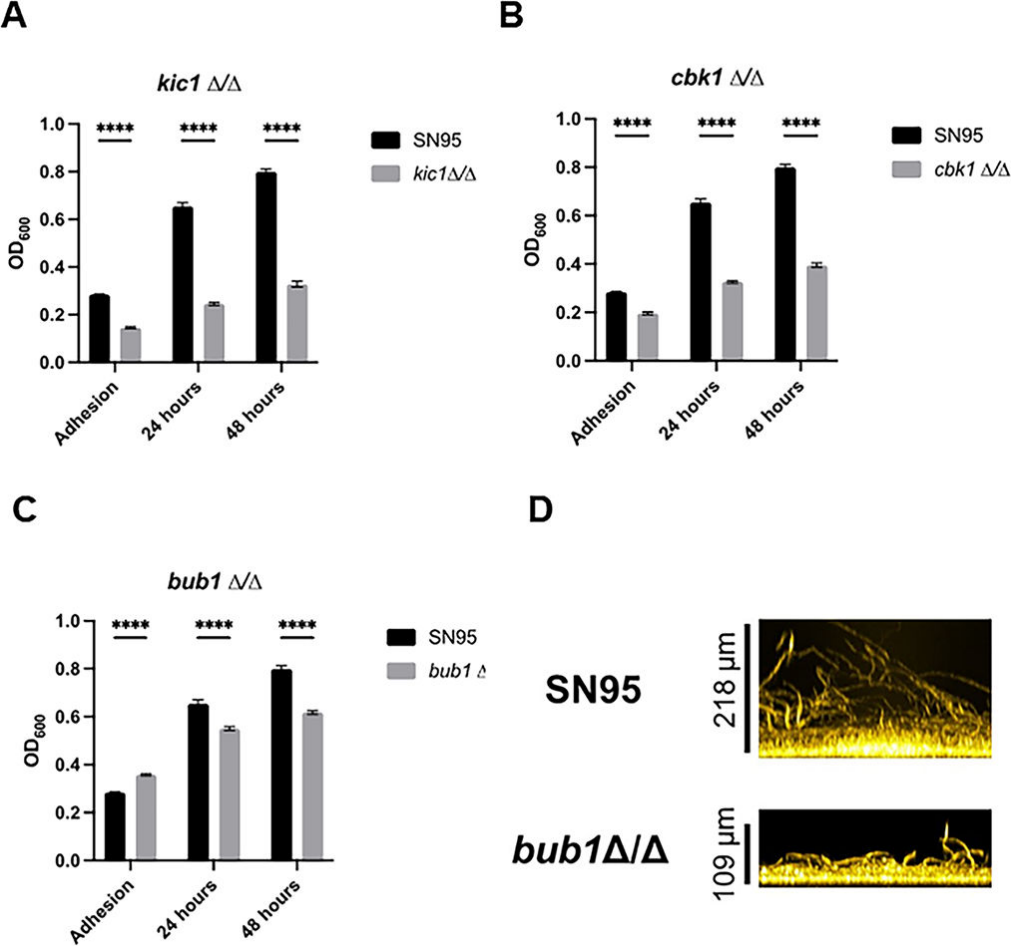
Confirmation of biofilm phenotypes in optical density assay. (**A–C**) The indicated strains were incubated in microtiter plates in RPMI medium at 37°C. For adhesion, the plates were washed with PBS at 90 minutes and the OD_600_ determined. Mature biofilms were measured at 24 hours and 48 hours. Error bars indicate the mean of 4–5 replicates with error bars standard deviation. **** indicates statistical significance (*P* < 0.05) by two-way ANOVA with correction for multiple comparisons. (**D**) Cross-sectional image of the *bub1*∆∆ and SN95 biofilms indicating that the *bub1*∆∆ mutant forms hyphae.

**Fig 8:**
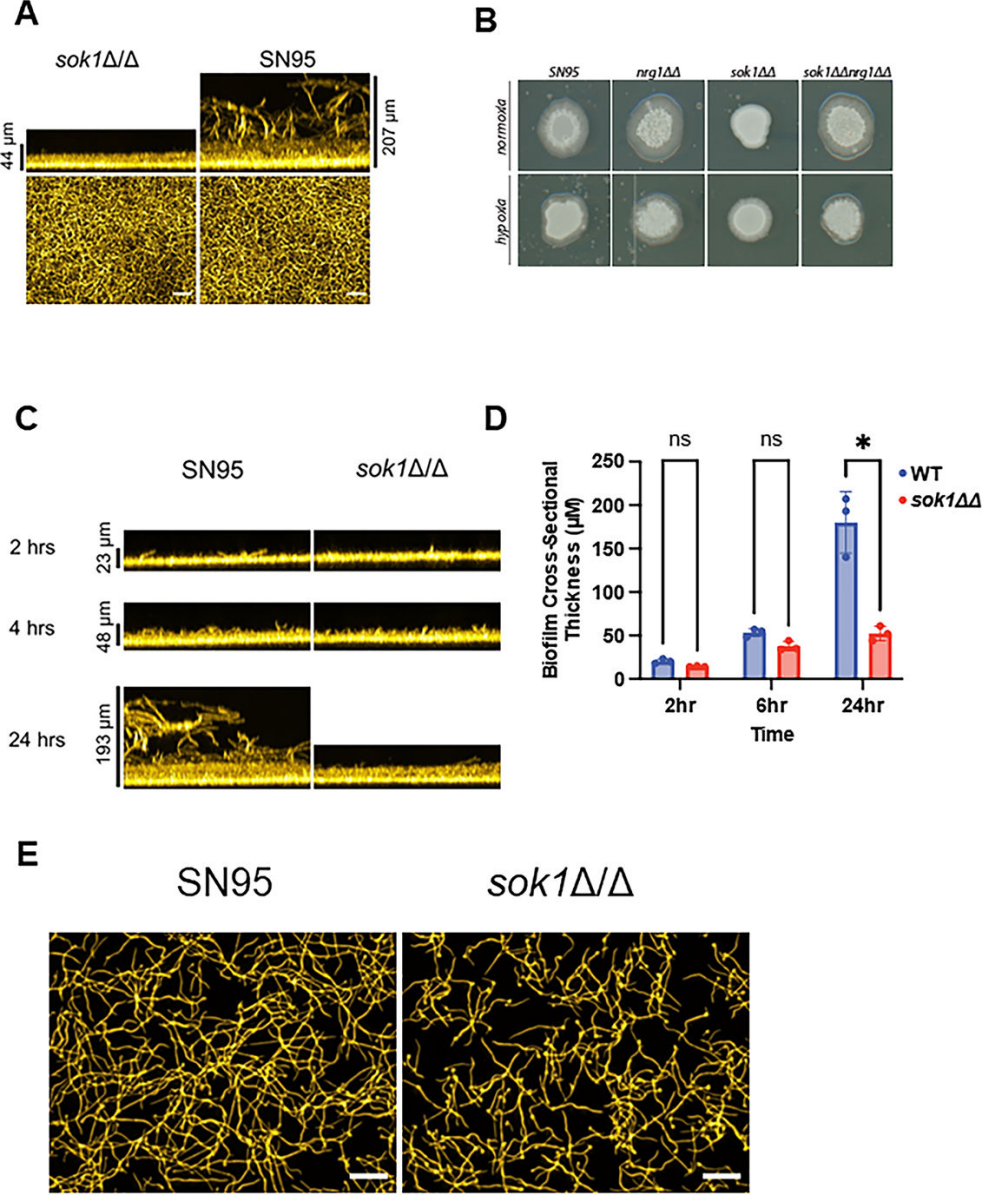
Sok1 is dispensable for filamentation at the basal biofilm layer and in hypoxia. (**A**) Cross-section and apical views of the biofilms formed by SN95 and the *sok1*∆∆ mutant in RPMI + 10% BCS at 37°C after 24 hours. (**B**) The indicated strains were spotted on RPMI agar plates supplemented with 10%BCS and incubated for 4 days in ambient air or a GasPak hypoxic environment. (**C**) Time course of biofilm formation with SN95 and *sok1*∆∆ strains. (**D**) Quantification of biofilm thickness over time course. Bars are means of three independent experiments with error bars indicating standard deviation. * indicates statistical significant (*P* < 0.05) difference between SN95 and *sok1*∆∆ mutant by two-tailed Student’s *t*-test. (**E**) Apical views of SN95 and *sok1*∆∆ mutant biofilms at 2 hours indicate filament formation in both samples.

We selected 12 mutants (Fig. 7A through C; Fig. S3) for testing using a third medium, RPMI at 37°C, and a quantitative microtiter plate-based biofilm density assay; the set included mutants from all three classes of phenotype severity. We characterized density after initial adhesion and at 24 hours and 48 hours. Regardless of the severity of initial screening phenotype, all of the tested mutants showed reduced biofilm densities at both 24 hours and 48 hours with none showing discordant phenotypes between the two time points. Seven of the 12 mutants had reduced initial adhesion with the RAM pathway kinase mutant *kic1*∆∆ having the most severe adhesion phenotype (Fig. 8A); mutation of the other RAM pathway kinase *CBK1* also led to reduced adhesion (Fig. 7B). Deletion of *BUB1*, one of the PKs with no filamentation defect (RPMI or RPMI + 10% BCS, 37°C), increased initial adhesion relative to SN95. However, the density of the biofilm was reduced at both 24 hours and 48 hours (Fig. 7C). The thickness of the biofilm is reduced in the *bub1*∆∆ mutant relative to SN95 (Fig. 7D) but hyphae extending from the basal layer of the biofilm are clearly visible. Thus, Bub1 is required for biofilm formation without dramatically affecting filamentation. In addition, we did not identify any significant growth defects for the *bub1*∆∆ mutant in any of the conditions we examined (Table S3). These data indicate that a large set of PKs contribute to *C. albicans* biofilm formation and, although many likely do so through their effect on filamentation, PKs must also regulate other processes that are required for this complex process to occur.

### Sok1 is dispensable for the establishment of the basal, filamentous layer of *C. albicans* biofilms *in vitro* but required for vertical extension of the biofilm

One of the PKs that has a very well-defined role in *in vitro* filamentation is Sok1 (44). At the initiation of filamentation, Sok1 phosphorylates the conserved transcriptional repressor Nrg1, leading to its degradation. This combined with reduced expression of *NRG1* is required for initiation of filamentation. Nrg1 represses the hypha transcriptional program and constitutive expression of *NRG1* locks the cells in yeast form. Consistent with previous literature, the *sok1*∆∆ mutant displayed a severe phenotype in all three conditions of our filamentation screen, and deletion of *NRG1* in the *sok1*∆∆ mutant restored filamentation to near wild-type levels, supporting the model that Sok1 is required for relief of Nrg1-mediated suppression of hyphae formation (44) (Fig. 5C; Fig. S4A).

As expected from its filamentation phenotype, the *sok1*∆∆ mutant showed reduced biofilm formation in RPMI + 10% BCS (Table S5). Imaging of the biofilm formed by the *sok1*∆∆ mutant shows a dense, narrow layer of cells with little of the vertical extension of hyphae seen in the WT biofilm (Fig. 8A). Surprisingly, the apical view of the biofilm formed by the *sok1*∆∆ mutant shows extensive hypha formation within this basal layer (Fig. 8A). These data suggest Sok1 is dispensable for initial hypha formation at the basal level of the biofilm. Previous studies by Upplapari et al. have shown that constitutive expression of *NRG1* prevents hyphae formation during biofilm-forming conditions, indicating the necessity for relief of Nrg1 repression for filamentation under these conditions (51). During filamentation, Sok1 is thought to contribute to the relief of Nrg1 repression (44).

These observations suggest that the specific environment encountered by *C. albicans* at the basal level of an *in vitro* biofilm triggers a Sok1-independent pathway leading to relief of Nrg1 hyphal repression. Our data and that reported by others indicate that the *sok1*∆∆ mutant fails to filament on semi-solid agar and in liquid conditions (Fig. 5C; Fig. S4A) (44). However, the *in vitro* biofilm is formed on a solid plastic substrate rather than on semi-solid agar or in liquid culture. To test whether adherence to plastic bypasses the requirement for Sok1 during filamentation, we incubated WT and *sok1*∆∆ strains in RPMIS medium at 37°C in 2 mL dishes containing a microscope coverslip. The slides were removed after 30 hours and the morphology of adhered cells was examined. The *sok1*∆∆ mutant cells adhered to the plastic of the coverslip but did not filament while WT cells formed robust hyphae and biofilm-like structures (Fig. S4B). Therefore, adherence to plastic does not appear to bypass the need for Sok1 during filamentation in the biofilm.

The environment of the *C. albicans* biofilm shows features of hypoxia (52) and we, therefore, hypothesized that *SOK1* may be dispensable for filamentation under hypoxic conditions. To test this hypothesis, we spotted the *sok1*∆∆ mutant on RPMI + 10% BCS agar plates and incubated them under hypha-inducing conditions (37°C) in ambient air or a hypoxic environment. As expected, the *sok1*∆∆ mutant did not show peripheral invasion in ambient air, and its invasion defect was suppressed in the *nrg1*∆∆ *sok1*∆∆ double mutant (Fig. 8B). Under hypoxic conditions, however, the *sok1*∆∆ mutant showed peripheral invasion that was similar to both WT and *nrg1*∆∆ mutants, indicating that *SOK1* is not required for filamentation under hypoxic conditions (Fig. 8B). Kowalski et al. have shown that oxygen gradients are established as fungal biofilms mature and that the oxygen content of the medium decreases with increasing distance from the air-liquid interface (53). We hypothesized that the low oxygen levels at the base of the biofilm may render *SOK1* dispensable for this early stage of biofilm formation.

To further characterize the effect of Sok1 on biofilm development, we compared the cross-sectional thickness of the WT and *sok1*∆∆ mutant biofilms at three time points (2 hours, 6 hours, and 24 hours; Fig. 8C). There was no difference in cross-sectional biofilm thickness between the WT strain and *sok1*∆∆ mutant at 2 hours or 6 hours but a large difference was present at 24 hours (Fig. 8D). Apical views of the biofilm at 2 hours also clearly show that the *sok1*∆∆ mutant forms hyphae at each time point (Fig. 8E). Consequently, it appears that the hyphae from the *sok1*∆∆ mutant are limited to regions relatively close to the base of the biofilm. Thus, Sok1 is not required for filamentation during the initial establishment of the basal layer of the biofilm but is required for the development of the vertical extensions beyond the basal layer. However, it seems unlikely that hypoxia is the primary reason for the ability of the *sok1*∆∆ mutant to filament at the basal layer of the biofilm because it seems unlikely that oxygen levels would be reduced fast enough to account for robust filamentation at 2 hours. Although in the *A. fumigatus* system, Kowalski et al. did not observe basal hypoxia until 12 hours of incubation (53).

Additional experiments will be required to definitely establish the molecular basis of this observation. However, our data strongly support a model in which two mechanisms mediate relief of Nrg1 repression during biofilm formation: a Sok1-independent mechanism operates at the initial basal stage of biofilm formation, and a Sok1-dependent mechanism that drives filament extension in the higher oxygen concentrations above the basal layer. We also have found that the *sok1*∆∆ mutant can filament under hypoxic conditions. However, it appears that these two features of the effect of Sok1 on filamentation are not directly related.

### The RAM pathway governs biofilm formation in an Ace2-independent manner that is partially bypassed by overexpression of *ALS3* and *HGC1 in vitro* and *in vivo*

The RAM pathway PKs (Cbk1 and Kic1) are established regulators of *C. albicans* biofilm formation and, accordingly, were among the most biofilm-deficient PK mutants in our screen (39). Gutierrez-Escribano et al. showed that Cbk1 phosphorylation of Bcr1 is required for biofilm formation *in vitro* and that this due, in part, to reduced expression of key biofilm-associated cell surface proteins such as Als3 (54). The transcription factor Ace2 is another bona fide Cbk1 substrate whose deletion mutant also has reduced biofilm formation (39). To determine whether Cbk1 phosphorylation of Ace2 was also involved in biofilm formation, we examined the density of biofilms formed by strains containing alleles of Ace2 lacking two or three of the Cbk1 consensus phosphorylation sites (55). Previous studies of these *ace2* phospho-site mutants showed that they had reduced cell separation and increased lateral yeast formation during filamentation (56). As shown in Fig. S4C, the adhesion, 24-hour, and 48-hour biofilms formed by these strains were not statistically different than WT, indicating that the contribution of Ace2 to biofilm formation is independent of Cbk1. This is consistent with our findings that Cbk1-dependent Ace2 functions appear to be mainly related to cell separation (55).

Heterologous expression of *ALS3* partially restores biofilm competency to the *bcr1*∆∆ mutant (56). Therefore, if the biofilm defects displayed by the *cbk1*∆∆ and *kic1*∆∆ mutants are primarily due to reduced Bcr1 activation, then the expression of *ALS3* from a heterologous promoter in the *cbk1*∆∆ and *kic1*∆∆ mutants should also restore biofilm formation to those mutants. An important distinction between *bcr1*∆∆ and *cbk1*∆∆/*kic1*∆∆ mutants, however, is that the former can filament in SC5314-derived strains (57) while the latter are unable to filament under conditions examined to date (39). As discussed above, filamentation is a central step in the formation of mature *C. albicans* biofilms. Therefore, the important roles that Cbk1 and Kic1 play in filamentation may be the feature of their function most important for biofilm formation. We, therefore, attempted to drive filamentation in the *kic1*∆∆ and *cbk1*∆∆ mutants by overexpression of the cyclin *HGC1*. Overexpression of *HGC1* is sufficient to drive filamentation in non-inducing conditions and can bypass the filamentation defects of mutants such as the *efg1*∆∆ mutant (58).

**Fig 9:**
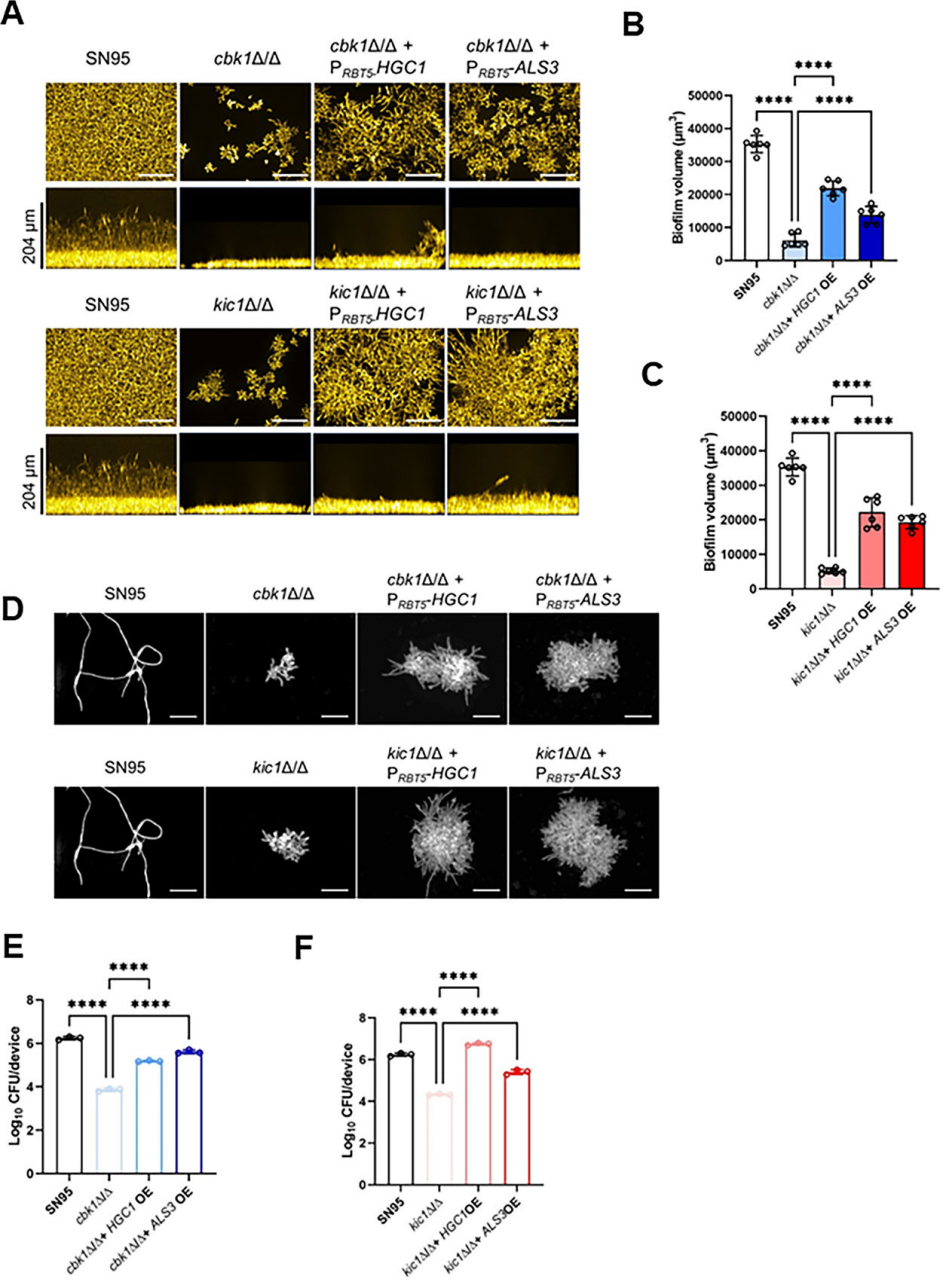
The role of Cbk1 in biofilm formation is independent of Ace2 and partially suppressed by overexpression of *ALS3* and *HGC1*. (**A**) Apical and cross-section images of 24-hour biofilms formed by the indicated strains in RPMI + 10% BCS at 37°C. Please note that the images of the SN95 control strain are duplicated to allow ease of comparison to both the *cbk1*∆∆ mutants (top panel) and *kic1*∆∆ mutants (bottom panel). (**B and C**) show a comparison of the biofilm volume for *cbk1*∆∆ (**B**) and *kic1*∆∆ (**C**) mutants and corresponding P*_RBT5_-ALS3*/*HGC1* overexpression strains. **** indicates a statistically significant difference between the indicated groups by two-way ANOVA with multiple comparisons test. (**D**) Fluorescence microscopy of Calcofluor-stained cells from biofilms for the indicated strains. (**E and F**) show the fungal burden of jugular venous catheters placed in rats that were infected with the indicated strains. The catheters were removed at 24 hours; the fungal cells were processed and plated as described in Materials and Methods. Bars indicate the mean of three replicates with standard deviation noted by error bars. **** indicates a statistically significant difference (*P* < 0.05) between groups by two-way ANOVA with multiple comparisons test. The fungal burden for *cbk1*∆∆ and *kic1*∆∆ mutants and corresponding P*_RBT5_-ALS3*/*HGC1* overexpression strains are shown in E and F, respectively.

We used the *RBT5* promoter (P*_RBT5_*) to drive the expression of *ALS3* and *HGC1* in the *cbk1*∆∆ and *kic1*∆∆ mutants using methods described by Mao et al. (59). Expression of the *RBT5* gene is strongly induced during biofilm formation and hypha formation in RPMI medium (59). Heterologous expression of *ALS3* and *HGC1* increased *in vitro* biofilm volume in both the *cbk1*∆∆ and *kic1*∆∆ mutants (Fig. 9A) although not to WT levels (Fig. 9B and C). Similar results were obtained for the *ALS3*- and *HGC1*-overexpressing *cbk1*∆∆ mutants using the optical density assay (Fig. S4D). Importantly, overexpression of *ALS3* and *HGC1* does not improve biofilm formation in the *ire1*∆∆ mutant (Fig. S4E), indicating that there is specificity to the heterologous *ALS3*/*HGC1* overexpression phenotypes.

Next, we imaged the biofilms at higher resolution to determine whether the overexpressing strains showed changes in cellular morphology. Indeed, *HGC1*^OE^ strains of both the *cbk1*∆∆ and *kic1*∆∆ mutants were elongated relative to the parental deletion mutants (Fig. 9D). By contrast, the *ALS3*^OE^ derivatives showed the round morphology of the parental strains. These data suggest that increased biofilm formation in the *HGC1*^OE^ strains may be due to increased filament-like cells while for *ALS3*^OE^ leads to morphology-independent partial rescue of biofilm formation in the RAM pathway. The ability of heterologous expression of *ALS3* and *HGC1* to rescue basal biofilm formation in the RAM PK mutants is not limited to *in vitro* conditions. Heterologous expression of both *ALS3* and *HGC1* in the *cbk1*∆∆ and *kic1*∆∆ mutants increased fungal burden in rat jugular catheters infected (60) with those strains relative to the parental strains (Fig. 9E and F).

### The HOG pathway is required for biofilm formation *in vivo*

**Fig 10:**
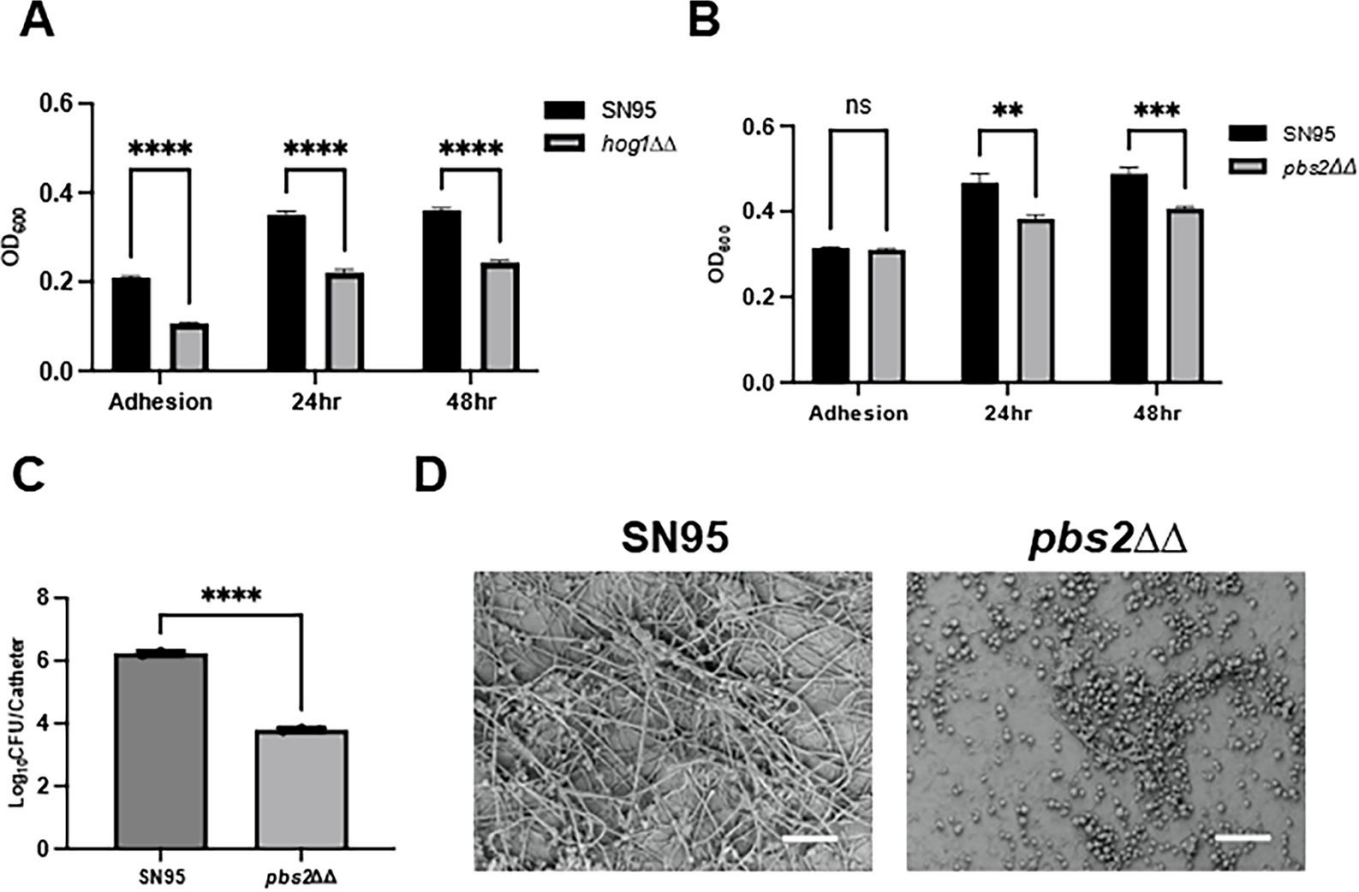
The HOG pathway plays a condition-dependent role in biofilm formation. The biofilm density for the *hog1*∆∆ (**A**) and *pbs2*∆∆ (**B**) mutants was determined by optical density in RPMI medium at 37°C at the indicated time points. Error bars indicate the mean of 4–5 replicates with error bars standard deviation. **** indicates statistical significance (*P* < 0.05) by two-way ANOVA with correction for multiple comparisons. (**C**) The fungal burden of jugular venous catheters placed in rats that were infected with either SN95 or the *pbs2*∆∆ mutant. The catheters were removed at 24 hours; the fungal cells were processed and plated as described in Materials and Methods. Bars indicate the mean of three replicates with standard deviation noted by error bars. **** indicates a statistically significant difference (*P* < 0.05) between groups by Student’s *t*-test. (**D**) Electron micrographs of biofilms formed in vascular catheters by SN95 and the *pbs2*∆∆ mutant.

The Mitchell lab previously found that glycerol homeostasis plays an important role in biofilm formation (61). As implied by its name, a central function of the HOG pathway is to regulate cellular levels of glycerol (36). In our primary screen, we found that deletion of Pbs2, the MAPKK of the HOG pathway, but not of the terminal MAPK Hog1, led to a minor defect in RPMI + 10% BCS biofilm formation but not in Spider medium (Table S5). We, therefore, decided to test both the *pbs2*∆∆ and *hog1*∆∆ mutants using our secondary assay to further explore the apparent condition-dependent role of the HOG pathway during biofilm formation. In RPMI medium without BCS, the *pbs2*∆∆ and *hog1*∆∆ mutants both showed reduced biofilm density (Fig. 10A and B) at 24 hours and 48 hours. The *hog1*∆∆ mutant also had significantly reduced initial adhesion but the *pbs2*∆∆ mutant did not. Desai et al. (61) found that the addition of glycerol to the medium suppresses the biofilm defect in other mutants that contribute to intracellular glycerol synthesis (e.g., the *rhr2*∆∆ mutant). The addition of glycerol to RPMI restored the density of the biofilm formed by the *pbs2*∆∆ mutant to wild-type levels (Fig. S4E). These data indicate that, under some *in vitro* conditions, the HOG pathway contributes to biofilm formation to a modest extent due, in part, to its role in glycerol production.

Because the HOG pathway showed condition-dependent effects on biofilm formation, we wondered whether the pathway was important in a model of clinically relevant biofilms. To test this possibility, we again used the rat vascular catheter model (5). We tested the *pbs2*∆∆ mutant instead of the *hog1*∆∆ mutant in this experiment because the *pbs2*∆∆ mutant does not have a defect in initial adhesion. As shown in Fig. 10C, the fungal density of the catheter biofilm formed by the *pbs2*∆∆ mutant was reduced by >2log_10_ CFU/catheter, a much stronger phenotype than expected based on the *in vitro* studies. We also compared the WT and *pbs2*∆∆ mutant biofilms using scanning electron microscopy (SEM). As expected, the WT biofilm was composed of an extensive network of hyphal structures with yeast phase cells and extracellular matrix scattered among it (Fig. 10D). By contrast, the *pbs2*∆∆ mutant biofilm is predominantly yeast-phase cells with extracellular matrix but few hyphal cells visible. The lack of filamentation of the *pbs2*∆∆ mutant in the rat catheter provides further evidence that the HOG pathway plays a condition-dependent role in *C. albicans* morphogenesis and biofilm formation.

## DISCUSSION

PKs are critically important regulators of a wide variety of biological processes that have been extensively studied in a variety of organisms and are currently one of the most important drug targets in medicine (62). Historically, the model yeast, *Saccharomyces cerevisiae*, played a seminal role in the development of our understanding of PK biology (63). To further the study of PKs in the common human fungal pathogen *C. albicans*, we have constructed a set of 99 PK and PK-related gene homozygous deletion mutants. Prior to this effort, other large-scale collections of PK strains have been reported in *C. albicans*. The initial collection was a transposon-derived library in the BWP17 background which has been specifically interrogated for mutants affecting cell wall processes as well as biofilm formation and filamentation (18). A PK deletion mutant library has also been reported for the reference strain SC5314 and has been the basis for a study of iron homeostasis as well as the kinase Ypk1 (19, 64). Finally, the characterization of a sub-library of PK mutants from the GRACE collection, a set of SC5314-derived strains in which ORFs are expressed from a doxycycline-regulated promoter (*tetO*) has been described recently and was used to study both filamentation and antifungal drug susceptibility (11, 65). Our overarching goal for creating this deletion set was to provide a genetic resource for the community to complement the transcription factor deletion set and the PK deletion sets that are available in other important human fungal pathogens.

The PK deletion mutant library described here has four features. First, it is in the SN background and, therefore, is complementary to the widely used TF deletion set (5) and a large-scale collection of other *C. albicans* deletion mutants (6). Second, it is amenable to further genetic manipulation through both auxotrophic and dominant drug selection markers. Third, the mutations are barcoded and are suitable for competitive fitness experiments using pools of mutants (6, 13, 14). Fourth, we provide benchmark phenotyping for the mutants over a range of commonly employed growth conditions for future reference in selecting mutants for study. As discussed previously, there are a number of essential PKs and the majority of the PKs for which we only obtained heterozygous mutants have indications that they are likely to be either essential or have significant fitness defects (Fig. 1C).

Our systematic characterization of this collection of PK mutants supports a general conclusion regarding the function of PKs in *C. albicans* biology and pathobiology. Specifically, the function of non-essential PKs and the phenotypic consequences of their corresponding deletion mutants are highly dependent on the specific environmental conditions under which the mutants are examined. For example, only two mutants, *mec1*∆∆ and *bud32*∆∆, showed growth phenotypes across all 10 conditions examined; correspondingly, Mec1 and Bud32 are likely to be involved in the fundamental process of DNA repair and RNA metabolism, respectively.

The environmental contingency of PK function is, however, most apparent during filamentation and biofilm formation, two process that are critical contributors to human disease. While all but 11 PK deletion mutants had an effect on filamentation under one of the six conditions we examined, one-third of the filament-associated PKs (30/88) affected filamentation on all six conditions while 15 mutants altered filamentation under a single condition. An example of the latter observation is the *tel1*∆∆ mutant which does not filament on Spider medium at 37°C but is similar to WT on the other conditions (Table S5). A pathway-level example supporting this conclusion is the HOG pathway which is classified in most review literature as repressing filamentation. This is based on observations of HOG pathway mutant behavior in liquid medium growth under non-hypha-inducing conditions (36). Our results are consistent with these observations in that we saw small number of hyphae formed by the *hog1*∆∆ mutant in YPD at 30°C (Fig. 5B); however, the previous reports used slightly different conditions and did not quantify the numbers. In contrast to these results, all three of the HOG PK mutants showed significantly reduced filamentation on solid media induction conditions (Fig. 4A and B). Furthermore, the *pbs2∆∆* mutant has a minor biofilm defect *in vitro* but a profound defect in an *in vivo* rat catheter biofilm model (Fig. 10B through D). The *in vivo* biofilm defect appears to be due to an inability of the *pbs2*∆∆ mutant to form filaments in the catheter. Our observations strongly support the conclusion that, under specific, infection-relevant conditions, the HOG pathway is involved in the positive regulation of filamentation while under other specific conditions it acts to suppress filamentation.

A second MAPK pathway for which we have found environmentally contingent functions during filamentation is the CWI. Previous work had demonstrated that the deletion mutant of the CWI pathway MAPK, *MKC1*, led to reduced filamentation and invasion on specific media when plated on solid agar (37). Furthermore, Kumamoto showed that contact with surfaces triggers the activation of the CWI pathway (38). In a liquid induction medium (YPD + 10% BCS), Xie et al. found that *PKC1* was required for filamentation but that the CWI pathway MAP kinases *BCK1*, *MKK2*, and *MKC1* were dispensable under these conditions. These observations were consistent with those of Kumamoto (38) who found that Mkc1 phosphorylation was reduced in the same liquid induction medium compared to conditions in which *C. albicans* was in contact with a solid or semi-solid substrate. We found that the CWI MAP kinase mutants had medium-dependent filamentation phenotypes of variable severity (Table S4) with the MAPKKK *bck1*∆∆ having the most severe phenotype on solid medium. In accordance with the results reported by Xie et al. (22), neither the *mkc1*∆∆ nor the *bck1*∆∆ mutants showed reduced filamentation in liquid RPMI + 1% BCS (Fig. 5C) but the filaments of the *mkc1*∆∆ mutant were significantly shorter than WT (Fig. 5D). It seems reasonable that cell wall remodeling during hyphal extension would require activation of the CWI pathway. This requirement for Mkc1 during hyphal elongation was not, however, observed *in vivo* (Fig. S1D and E). It is possible that the reduction in length observed *in vitro* may be due to a difference in the timing of the analysis and if earlier time points were used *in vivo* then a difference in the rate of filament lengthening might be observable.

The synthetic genetic interactions of CWI and HOG pathway PKs with *NRG1* emphasized that repression of the hyphal transcriptional program cannot be the only function of Nrg1. Under most *in vitro* induction conditions, Nrg1 protein expression is restored as hyphal morphogenesis continues (44, 45). Because Nrg1 regulates the expression of non-hyphal genes and other TFs, it seems likely that Nrg1 may play additional roles after initiation of hyphal morphogenesis (42). Our genetic interaction data with CWI pathway mutants suggest it may participate with CWI PKs in the maintenance of hyphal extension. These potential non-canonical roles for Nrg1 may be related to results from the Mitchell lab (66) as well as our own (67) describing Nrg1-related phenotypes and functions that are distinct from those expected based on its canonical function as an on-off switch for *C. albicans* filamentation.

Our analysis of the RAM pathway during biofilm formation also provides insights into the roles of filamentation during this process (39). Filamentation is not only a separate morphological state but is accompanied by a dramatic change in the expression of a variety of genes; indeed, many of these genes are typically expressed only in hyphae and play roles in the ability of *C. albicans* to form hyphae (41, 42). Cbk1 phosphorylates Bcr1 to activate the expression of hypha- and biofilm-associated genes such as *ALS3* (54). Overexpression of *ALS3* in *bcr1*∆∆, *cbk1*∆∆, or *kic1*∆∆ mutants partially suppress their biofilm defects further supporting the important function of the Kic1/Cbk1-Bcr1-Als3 circuit during biofilm formation (Fig. 9A through D). This is likely due to increased intracellular or cell substrate adhesion mediated by the cell wall protein Als3 because there is no change in the morphology of the *ALS3*^OE^ derivatives of the *cbk1*∆∆ or *kic1*∆∆ mutants (Fig. 9D).

By contrast, overexpression of *HGC1* in the *cbk1*∆∆ and *kic1*∆∆ mutants led to increased filament-like cells relative to the parental strains (Fig. 9D). Hgc1 is a cyclin that functions with Cdc28 to phosphorylate key proteins required for *C. albicans* polarized growth during filamentation (68). Cdc28/Hgc1 also phosphorylates the RAM network component Mob2 (69). Mob2, in turn, is required for Cbk1 function (39). To our knowledge, deletion of any RAM pathway mutant prevents initiation of filamentation under any condition reported in the literature. Therefore, the ability of *HGC1* overexpression to increase filamentation in biofilms of the *cbk1*∆∆ and *kic1*∆∆ mutants cannot result from a linear Cdc28/Hgc1-Mob2-Cbk1 pathway. Instead, this result indicates that *HGC1* overexpression bypasses this requirement for filamentation, presumably through a mechanism that functions downstream of the RAM pathway. These filamentous cells do not, however, extend above the basal layers as do WT cells, further emphasizing the conclusion that they are not completely normal hyphae.

We have also discovered that the development of the hyphal extensions from the basal layer of *C. albicans* biofilms involves an environmentally contingent function of Sok1. Previous genetic studies have linked Sok1 to the process of relieving Nrg1 repression and, consequent, initiation of filamentation (44). Although our results with the *sok1*∆∆ mutant under standard filamentation conditions were completely consistent with this model (Fig. 5C; Fig. S4A), we observed filamentation of the *sok1*∆∆ mutant during biofilm formation (Fig. 8). Further analysis indicated that the filamentation was restricted to the basal layer of the biofilm and that the profound filamentation defect of the *sok1*∆∆ mutant could be suppressed under hypoxic conditions (Fig. 8B). However, it is unlikely that hypoxia bypasses the requirement for Sok1 during establishment of the biofilm basal layer based on our time course experiments. Once again, we find that the regulation of *C. albicans* filamentation employs a variety of condition-dependent regulators. Further work will be required to understand the molecular mechanisms of this dramatic example of environmentally contingent regulation of *C. albicans* filamentation and biofilm formation.

As a resource, we hope that this collection will facilitate a more detailed understanding for the role of PKs in the regulation of *C. albicans* biology. Considering the extensive number of PKs that affect filamentation under at least one *in vitro* condition, the identification of specific targets or sets of targets that mediate the downstream functions of the PKs during filamentation will be required before such an understanding can be achieved. Integrating this genetic resource with existing, and future, phosphoproteomic analyses of the filamentation process as reported by Wilger et al. (70), Cao et al. (71), and Min et al. (72) are likely to lead to new insights into the specific targets required for filamentation.

As a brief illustration of the hypothesis-generating potential of such an approach, we consider the RAM PK Cbk1 (39), which is required for filamentation in all *in vitro* conditions as well as in our *in vivo* assay. The two most widely studied substrates of Cbk1, the TFs Ace2 and Bcr1, play condition-dependent roles in morphogenesis and, thus, cannot account for the profound and consistent filamentation defects displayed by *cbk1*∆∆ mutants (55, 56, 58). Interestingly, the deletion of *NRG1* in the *cbk1*∆∆ background partially suppresses its lack of filamentation in a manner that appears to involve its canonical substrate Ssd1, an RNA-binding protein (73). However, neither the *nrg1*∆∆ *cbk1*∆∆ nor the *ssd1*∆∆ *cbk1*∆∆ double mutant forms true hyphae (73), indicating that Cbk1 has additional hyphae-associated substrates. The phosphoproteomic data set collected during *C. albicans filamentation* by Min et al. (72) showed that 58 proteins are phosphorylated at a Cbk1 consensus motif. GO term analysis revealed that this set of potential Cbk1 substrates is enriched for proteins involved in filamentous growth (16/58, FDR 0.01, Benjamini correction) and Swe1 and Bck1, two PKs required for filamentation. Thus, the combination of functional genetic data from the PK collection with phosphoproteomic data derived from a specific *C. albicans* phenotype can rapidly generate hypotheses regarding the mechanisms of PK-mediated regulation.

The integration of phosphoproteomic data with functional data based on PK genetic experiments is also facilitated by multiple genetic resources for the study of *C. albicans* PKs now available. Our collection provides a set of PK mutants in a strain background amenable to further genetic manipulation and the ability to used barcode-facilitated pooled genetic screens. A similar deletion set has been generated by the Morschhauser lab in the widely used reference strain SC5314 (19). This set fits well with the extensive use of the SC5314 strain in virulence studies and avoids potential complications in the interaction of mutations with auxotrophic markers. Finally, strains containing tetracycline-regulated PK alleles as the only source of the PKs are a subset of the larger GRACE collection and have been used to study PK function as well. The tetracycline-regulated alleles allow the study of essential PKs or low-fitness PK mutants and are also well validated in mouse models of infection. Two limitations of the tetracycline-regulated PK strains are that in the absence of doxycycline, the alleles tend to be over-expressed and complete suppression of expression may not be achieved in the presence of doxycycline. The latter limitation is most likely the reason for distinctions in some of the PK filamentation phenotypes found by Lee et al. and this work (11).

One of the themes that emerge from this work is that it is difficult to make general conclusions regarding the contribution of PKs to complex phenotypes such as filamentation and biofilm formation. These limitations apply to the non-screening experiments we present herein as well. For example, our conclusion that, in RPMI medium, phosphorylation of Ace2 by Cbk1 is dispensable for biofilm formation may not apply to other conditions. Furthermore, it is possible that Ace2 is phosphorylated by Cbk1 but, that in RPMI medium, loss of Cbk1-dependent Ace2 function leads to a compensatory response that suppresses the phenotype. Similarly, the timing of a given PK function relative to relief of Nrg1 repression during filamentation is also likely to vary from one condition to another and our conclusions based on a single *in vitro* filament induction condition need to be viewed in light of such caveats.

In summary, the environmentally contingent nature of PK function suggests that the analysis of complex phenotypes such as filamentation and biofilm formation requires examination of multiple conditions and, as others have also emphasized, that care must be taken when generalizing the results to other niches and environments in the absence of direct experimentation. In this work, we examined only three media and two different temperatures; therefore, different results are to be expected if other conditions are employed. *C. albicans* adapts to a wide range of environmental niches and PKs mediate important sensing and signaling networks. Consequently, this complexity and variation in function should not be surprising. Indeed, the extensive network of PKs that affect *C. albicans* filamentation is likely due to the fact that filamentation and the related pathobiological process, biofilm formation, are an integral part of its physiology and, thereby, dependent upon the function of a wide variety of other physiologic processes and systems.

## MATERIALS AND METHODS

### General culture methods

All media was prepared using previously reported recipes (5). Unless otherwise indicated, *C. albicans* strains were pre-cultured prior to experiments by inoculation of a colony from an agar plate into yeast peptone dextrose (YPD) and incubation overnight at 30°C. Library PK mutants that had been genotypically confirmed were stored in YPD-glycerol stocks at −80°C and recovered by streaking on a YPD agar plate followed by incubation at 30°C for 2 days.

### Protein kinase gene deletion library construction

All known/predicted protein kinase genes (Tables S1 and S2) were deleted using modified CRIME (CRISPR-Cas9-induced marker excision) approach enabling marker recycling through a direct repeat mediated crossover (21, 74). The 3′ portion of the split cassette was amplified from the pMH03 plasmid using a universal *LEU2* forward primer (OL48, Table S1) and a reverse primer with a pMH03 specific sequence followed by an 80nt portion of the 3′ UTR adjacent to the stop codon. 5′ portion of the deletion cassette was modified to include a barcode at the 5′ end. To this end, a set of modified 5′ split cassette plasmids (pMH04) was created bearing the 48 barcodes used previously by Homann et al. (5) preceded by a common adapter sequence. The 5′ portion of the split cassette was then amplified from a pMH04-derived barcoding plasmid using a primer with 80nt target gene start codon proximal sequence followed by the adapter sequence and a universal *LEU2* marker reverse primer (OL49, Table S2). For each protein kinase gene, two independent sgRNAs were designed using the ChopChop tool. The sgRNA and Cas9 expression cassettes were prepared as described in reference 68 and co-transformed into *C. albicans* SN152 strain together with the split deletion cassette (21). Transformants were selected on CSM-Leu media. Correct integration of the deletion cassette was verified by PCR and the absence of amplicon from the targeted ORF.

### Construction of fluorescently labeled strains

Fluorescently labeled SN95 was generated with the p*ENO1-NEON-NAT1* and the *mkc1*∆∆ mutant was labeled with p*ENO1*-iRFP-*NAT1* plasmid as previously described (47) and transformants were selected on YPD containing 200 µg/mL nourseothricin. Fluorescent isolates were confirmed by flow cytometry. The resulting His^−^ strains were converted to His^+^ by integrating plasmid pGEM-*HIS1* as previously described (75). The fluorescently NEON-labeled SN95 showed no difference in fitness relative to the SN95 parent in YPD at 30°C.

### Construction of *NRG1* deletion mutants

Strains lacking *NRG1* in the SN95 and the PK mutant strains indicated in Fig. 5 were generated by CRISPR-Cas9-mediated disruption using gRNA and repair constructs reported previously (66).

### Construction of P*_RBT5_-ALS3* and P*_RBT5_-HGC1* strains

To generate homozygous strains overexpressing hypha-specific cyclin gene, *HGC1* and adherence gene, *ALS3*, a P*_RBT5_* cassette (58) containing flanking homology to the gene upstream promoter region and nourseothricin resistant gene region was amplified using primer sets *HGC1* OE extension/F and *HGC1* OE extension/R, *ALS3* OE extension/F, and *ALS3* OE extension/R from YM160 strain (SC5314 P_RBT5_-*HGC1*) and AB55 strain (SC5314 P_RBT5_-*ALS3*) gDNA.

The sgRNA cassettes for the 5′ regions of each gene were generated using split-joint PCR using primers HGC1p sgRNA/F and HGC1p SNR52/R, ALS3p sgRNA/F and ALS3p SNR52/R. The constructs and guide RNAs were then transformed into *cbk1*Δ/Δ, *kic1*Δ/Δ and *ire1*∆∆ mutant strains. Transformants were selected on YPD + nourseothricin medium and correct integration was confirmed by PCR.

### Flow cytometry competitive fitness assay

Competitive fitness assays were performed in 96-well plates containing 200 µL of medium per well. Wells were inoculated with individual strains to be tested and the reference strain SN95-mNeonGreen and grown statically overnight at 30°C. Dilutions were performed to standardize input of the unlabeled protein kinase mutant library strains and SN95-mNeonGreen in a 1:1 ratio, at 2 × 10^4^ cells/mL final concentration in varying growth conditions, as indicated. Co-cultures were grown at 30°C or 37°C for 24 hours in ambient air before analyzing on an Attune NxT Flow Cytometer with CytKick autosampler. The gating strategy was optimized for single cells. mNeonGreen positive and negative populations were identified by histogram plot with 100,000 cells counted per sample. The percent of mNeonGreen negative cells in experimental growth medium conditions was divided by the percent of mNeonGreen negative cells at 30°C in YPD to determine competitive fitness. Three biological replicates of each condition were performed and the difference between mutant and SN95 was analyzed for statistical significance by two-sided Student’s *t*-test (*P* < 0.05) in Microsoft Excel. Experimental growth media were as follows: YPD, YP + 2% galactose, YP + 2% glycerol, YP + 2% acetate, YPD + 150 mM HEPES (pH 4), YPD + 150 mM HEPES (pH 9), YPD + 1.5 M NaCl, YPD + 1.5 M sorbitol, YNB + arg + 1% glucose + AmSO_4_, YPD + 0.000407% SDS. All were screened at 30°C, with the exception of YPD at both 30°C and 37°C. Average competitive fitness scores were used to generate a heat map in Morpheus (https://software.broadinstitute.org/morpheus), hierarchically clustered by growth condition and strain with one minus Pearson correlation, average linkage.

### Spot dilution assays

Cells from overnight cultures were washed twice with PBS prior to quantifying OD_600_. Strains were diluted to an OD_600_ of 1, followed by 10-fold serial dilutions. Three microliters from each dilution was spotted on agar plates and grown inverted at 30°C or 37°C as indicated for 24 hours on YPD, YP + 2% galactose, YP + 2% glycerol, YP + 2% acetate, YPD + 1.5 M sorbitol, or YPD + 1.5 M NaCl. Images were acquired at 24 hours and are representative of triplicates.

### Agar plate-based screening assay of filamentation phenotypes

Overnight cultures of PK mutants were diluted to 0.1 OD_600_ and 5 µL was spotted on YPD, Spider medium, RPMI, or RPMI + 10% bovine calf serum plates; SN95 control strains were also spotted on each plate. The plates were incubated at 30°C or 37°C. Peripheral invasion was scored at day 5 using the scale shown in Fig. S1A for mutants with reduced filamentation. For mutants with increased filamentation, they were scored at the time point when the mutant showed increased filamentation relative to SN95. The screen was performed in biological duplicate. For experiments performed under hypoxia, the plates were placed in a GasPak and incubated as described above.

### *In vitro* liquid culture-based filamentation assay

For *in vitro* hyphal induction in liquid media, *C. albicans* strains were incubated overnight at 30°C in YPD media, harvested, and diluted into RPMI supplemented with either 1% or 10% BCS at a 1:50 ratio and incubated at 37°C for 4 hours. Induced cells were fixed with 1% (vol/vol) formaldehyde. Fixed cells were then imaged using the Echo Rebel upright microscope with a 60× objective; pseudohyphae were defined as filamentous cells with non-parallel cell walls and hyphae were defined as filamentous cells with parallel cell walls. The assays were conducted in biological triplicates on different days. Differences in the distribution of morphotypes were analyzed using data pooled from independent replicates and Chi-squared test (*P* < 0.05). The length of the hyphae was determined as previously described.

### Imaging assay of *in vivo* filamentation

The imaging of dBA/2 mice ears infected with fluorescently labeled SN95 (NEON) and mkc1∆∆ mutant (iRFP) was carried out as described previously (46). Each ear (*n* = 3) was inoculated with a 1:1 mixture of SN95:*mkc1*∆∆ mutant. The ears were imaged 24 hours post-inoculation. Multiple Z stacks (minimum 15) were used to score the yeast vs filamentous ratio. The cells were considered as a “yeast” if the cells were round and/or budded cells. Furthermore, yeast cells were required not to project through multiple Z stacks. The cells were considered “filamentous” if the cells contained an intact mother and filamentous which was at least twice the length of the mother body. A minimum of 100 cells from multiple fields were scored. Paired Student’s *t-*test with Welch’s correction (*P* > 0.05) was used to define the statistical significance which was carried out using GraphPad Prism software. The filament length of the *in vivo* samples was measured as described previously (46). Briefly, a Z stack image of the reference or mutant strain was opened in ImageJ software and the distance between the mother neck to the tip of the filament was measured. At least 50 cells per strain from multiple fields were measured. Statistical significance was determined by the Mann-Whitney U test (*P* > 0.05).

### Imaging-based screening of PK mutant biofilm formation

As previously described (51), strains were inoculated into 96-well plates at an OD_600_ of 0.05 in 100 µL Spider medium or RPMI + 10% BCS. The wells were incubated at 37°C for 90 min with shaking (60 rpm) to adhere cells. The wells were then carefully washed twice with PBS. Fresh medium (100 µL) was added to each well and returned to the incubator for 24 hours with shaking. The medium was carefully aspirated and the biofilms were fixed with 4% formaldehyde in PBS (100 µL) for 1 hour; washed with PBS and then treated with Calcofluor White (200 µg/mL) overnight with shaking (60 rpm). The wells were then sequentially treated with 50% and then 100% thiodiethanol solution for 1 hour. Images were collected using a Keyence BZ-X800E microscope. Processing of images was performed as described in Shama et al. (51). Each mutant was screened in biological duplicate and compared to SN95 biofilms formed contemporaneously.

### Optical density-based assay of biofilm formation

Biofilm growth was assayed as described in Glazier et al. (76). Briefly, strains were grown overnight at 30°C in liquid YPD with shaking, washed twice in phosphate-buffered saline (PBS), diluted to an OD_600_ of 0.5 in desired media. 200 µL of the suspension was dispensed into six wells of a 96-well plate (Corning Incorporated 96-well plate, catalog no. 3596). The cells were then incubated in a 37°C incubator for 90 minutes to allow adherence. Next, the media was removed, and the wells were washed once with PBS to remove non-adhered cells. Adherence was measured OD_600_ using a SpectraMax plate reader. The biofilm-inducing media was replaced and cells were further incubated at 37°C, without shaking. After 24 hours or 48 hours, the media were aspirated; the cells were washed with PBS as above and the OD_600_ was measured. Three biological replicates with six technical replicates per strain were analyzed for each strain and the experiment was repeated independently 2–3 times. Differences between strains were analyzed by ANOVA and multiple comparison correction with statistical significance set at an adjusted *P* value < 0.05.

### Rat model of *Candida albicans* vascular catheter infection

*In vivo C. albicans* biofilms were studied using an external jugular-vein, rat-catheter infection model as previously described (59). Briefly, a 1 × 10^6^  cells/mL inoculum for each strain or strain combination was allowed to grow on an internal jugular catheter placed in a pathogen-free female rat (16-week-old, 400  g) for 24 hours. The catheter volumes were then removed and the catheters were flushed with 0.9% NaCl. The biofilms were dislodged by sonication and vortexing. Viable cell counts were determined by dilution plating. Three replicates were performed for each strain. Differences in fungal burden were analyzed by ANOVA and correction for multiple comparisons with a significant difference defined as a *P* < 0.05. After a 24-hour biofilm formation phase, the devices were removed, sectioned to expose the intraluminal surface, and processed for SEM imaging. Briefly, 1 mL fixative (4% formaldehyde and 1% glutaraldehyde in PBS) was added to each catheter tube and tubes were fixed at 4°C overnight. Catheters were then washed with PBS prior to incubation in 1% OsO4 for 30 min. Samples were then serially dehydrated in ethanol (30%–100%). Critical point drying was used to completely dehydrate the samples prior to palladium-gold coating. Samples were imaged on an SEM LEO 1530, with Adobe Photoshop 2022 (v. 23.2.2) used for image compilation.

### Phospho-Mkc1 western blot

Western blotting was performed as described previously (77, 78). Briefly, harvested cell pellets were resuspended in 300–500 µL of lysis buffer (50 mM Tris-HCl [pH 7.5], 150 mM NaCl, 1 mM EDTA, 1% Triton X-100) supplemented with protease and phosphatase inhibitor cocktail (Thermo Scientific). The suspension was then transferred to a tube with glass beads and lysed by 10 30-s bursts using a bead beater. The resulting lysate was cleared by centrifugation and total protein was determined by Bio-Rad protein assay dye concentrate according to the manufacturer’s instructions. 50 µg of the lysate was separated on a 10% Mini-Protean TGX gel (Bio-Rad) and transferred to a nitrocellulose membrane. Membranes were blocked for 60 minutes at room temperature in 5% BSA in 50 mM Tris (pH 7.5), 150 mM NaCl, 0.01% Tween 20 (TBST). The membranes were then probed with anti p44/p42 MAPK Thr202/Tyr204 antibody (Cell Signaling) followed by anti-rabbit horseradish peroxidase (HRP)-conjugated secondary antibody (Bio-Rad). Blots were imaged on a myECL imager (Thermo Scientific) using a SuperSignal West Femto ECL reagent (Thermo Scientific).

## SUPPLEMENTAL MATERIAL

Fig. S1 mbio.01249-24-s0001.tif Filamentation phenotypes.

Fig. S2 mbio.01249-24-s0002.tif Biofilms for all 99 PK mutants.

Fig. S3 mbio.01249-24-s0003.tif Biofilm density data for selected mutants.

Fig. S4 mbio.01249-24-s0004.tif Biofilm phenotypes of RAM pathway and SOK1 mutants.

Legends mbio.01249-24-s0005.docx Legends for supplemental material.

Table S1 mbio.01249-24-s0006.xlsx All protein kinases targeted.

Table S2 mbio.01249-24-s0007.xlsx Putatively essential PKs for which only heterozygous mutants were obtained.

Table S3 mbio.01249-24-s0008.xlsx Competitive fitness data for all PK mutants on growth conditions.

Table S4 mbio.01249-24-s0009.xlsx Summary of PK mutants with growth phenotypes on 10 conditions.

Table S5 mbio.01249-24-s0010.xlsx Summary of filamentation and biofilm phenotypes for all mutants.

ASM does not own the copyrights to Supplemental Material that may be linked to, or accessed through, an article. The authors have granted ASM a non-exclusive, world-wide license to publish the Supplemental Material files. Please contact the corresponding author directly for reuse.

## References

[1] Proctor DM, Drummond RA, Lionakis MS, Segre JA. 2023. One population, multiple lifestyles: commensalism and pathogenesis in the human mycobiome. Cell Host Microbe 31:539–553. doi:10.1016/j.chom.2023.02.010 37054674 PMC10155287

[2] Pappas PG, Lionakis MS, Arendrup MC, Ostrosky-Zeichner L, Kullberg BJ. 2018. Invasive candidiasis. Nat Rev Dis Primers 4:18026. doi:10.1038/nrdp.2018.26 29749387

[3] Calderone RA, ed. 2002. Candida and candidiasis. American Society of Microbiology, Washington D.C.

[4] Bruno VM, Mitchell AP. 2004. Large-scale gene function analysis in Candida albicans. Trends Microbiol 12:157–161. doi:10.1016/j.tim.2004.02.002 15051065

[5] Homann OR, Dea J, Noble SM, Johnson AD. 2009. A phenotypic profile of the Candida albicans regulatory network. PLoS Genet 5:e1000783. doi:10.1371/journal.pgen.1000783 20041210 PMC2790342

[6] Noble SM, French S, Kohn LA, Chen V, Johnson AD. 2010. Systematic screens of a Candida albicans homozygous deletion library decouple morphogenetic switching and pathogenicity. Nat Genet 42:590–598. doi:10.1038/ng.605 20543849 PMC2893244

[7] Schwarzmüller T, Ma B, Hiller E, Istel F, Tscherner M, Brunke S, Ames L, Firon A, Green B, Cabral V, et al.. 2014. Systematic phenotyping of a large-scale Candida glabrata deletion collection reveals novel antifungal tolerance genes. PLoS Pathog 10:e1004211. doi:10.1371/journal.ppat.1004211 24945925 PMC4063973

[8] Liu OW, Chun CD, Chow ED, Chen C, Madhani HD, Noble SM. 2008. Systematic genetic analysis of virulence in the human fungal pathogen Cryptococcus neoformans. Cell 135:174–188. doi:10.1016/j.cell.2008.07.046 18854164 PMC2628477

[9] Lee KT, Hong J, Lee DG, Lee M, Cha S, Lim YG, Jung KW, Hwangbo A, Lee Y, Yu SJ, Chen YL, Lee JS, Cheong E, Bahn YS. 2020. Fungal kinases and transcription factors regulating brain infection in Cryptococcus neoformans. Nat Commun 11:1521. doi:10.1038/s41467-020-15329-2 32251295 PMC7090016

[10] Furukawa T, van Rhijn N, Fraczek M, Gsaller F, Davies E, Carr P, Gago S, Fortune-Grant R, Rahman S, Gilsenan JM, Houlder E, Kowalski CH, Raj S, Paul S, Cook P, Parker JE, Kelly S, Cramer RA, Latgé J-P, Moye-Rowley S, Bignell E, Bowyer P, Bromley MJ. 2020. The negative cofactor 2 complex is a key regulator of drug resistance in Aspergillus fumigatus. Nat Commun 11:427. doi:10.1038/s41467-019-14191-1 31969561 PMC7194077

[11] Lee Y, Hossain S, MacAlpine J, Robbins N, Cowen LE. 2023. Functional genomic analysis of Candida albicans protein kinases reveals modulators of morphogenesis. iScience 26:106145. doi:10.1016/j.isci.2023.106145 36879823 PMC9984565

[12] Robbins N, Ketela T, Kim SH, Cowen LE. 2023. Chemical-genetic approaches for exploring mode of action of antifungal compounds in the fungal pathogen Candida albicans. Methods Mol Biol 2658:145–165. doi:10.1007/978-1-0716-3155-3_10 37024700 PMC11019913

[13] Witchley JN, Penumetcha P, Abon NV, Woolford CA, Mitchell AP, Noble SM. 2019. Candida albicans morphogenesis programs control the balance between gut commensalism and invasive infection. Cell Host Microbe 25:432–443. doi:10.1016/j.chom.2019.02.008 30870623 PMC6581065

[14] Pérez JC, Kumamoto CA, Johnson AD. 2013. Candida albicans commensalism and pathogenicity are intertwined traits directed by a tightly knit transcriptional regulatory circuit. PLoS Biol 11:e1001510. doi:10.1371/journal.pbio.1001510 23526879 PMC3601966

[15] Wakade RS, Ristow LC, Wellington M, Krysan DJ. 2023. Intravital imaging-based genetic screen reveals the transcriptional network governing Candida albicans filamentation during mammalian infection. Elife 12:e85114. doi:10.7554/eLife.85114 36847358 PMC9995110

[16] Koselny K, Green J, Favazzo L, Glazier VE, DiDone L, Ransford S, Krysan DJ. 2016. Antitumor/antifungal celecoxib derivative AR-12 is a non-nucleoside inhibitor of the ANL-family adenylating enzyme acetyl CoA synthetase. ACS Infect Dis 2:268–280. doi:10.1021/acsinfecdis.5b00134 27088128 PMC4828684

[17] Nobile CJ, Fox EP, Nett JE, Sorrells TR, Mitrovich QM, Hernday AD, Tuch BB, Andes DR, Johnson AD. 2012. A recently evolved transcriptional network controls biofilm development in Candida albicans. Cell 148:126–138. doi:10.1016/j.cell.2011.10.048 22265407 PMC3266547

[18] Blankenship JR, Fanning S, Hamaker JJ, Mitchell AP. 2010. An extensive circuitry for cell wall regulation in Candida albicans. PLoS Pathog 6:e1000752. doi:10.1371/journal.ppat.1000752 20140194 PMC2816693

[19] Ramírez-Zavala B, Krüger I, Dunker C, Jacobsen ID, Morschhäuser J. 2022. The protein kinase Ire1 has a Hac1-independent essential role in iron uptake and virulence of Candida albicans. PLoS Pathog 18:e1010283. doi:10.1371/journal.ppat.1010283 35108336 PMC8846550

[20] Noble SM, Johnson AD. 2005. Strains and strategies for large-scale gene deletion studies of the diploid human fungal pathogen. Eukaryot Cell 4:298–309. doi:10.1128/EC.4.2.298-309.2005 15701792 PMC549318

[21] Huang MY, Mitchell AP. 2017. Marker recycling in Candida albicans through CRISPR-Cas9 induced marker excision. mSphere 2:e00050-17. doi:10.1128/mSphere.00050-17 28317025 PMC5352831

[22] Xie JL, Grahl N, Sless T, Leach MD, Kim SH, Hogan DA, Robbins N, Cowen LE. 2016. Signaling through Lrg1, Rho1, and Pkc1 governs Candida albicans morphogenesis in response to diverse cues. PLoS Genet 12:e1006405. doi:10.1371/journal.pgen.1006405 27788136 PMC5082861

[23] Park H, Liu Y, Solis N, Spotkov J, Hamaker J, Blankenship JR, Yeaman MR, Mitchell AP, Liu H, Filler SG. 2009. Transcriptional responses of Candida albicans to epithelial and endothelial cells. Eukaryot Cell 8:1498–1510. doi:10.1128/EC.00165-09 19700637 PMC2756863

[24] Bruckmann A, Künkel W, Härtl A, Wetzker R, Eck R. 2000. A phosphatidylinositol 3-kinase of Candida albicans influences adhesion, filamentous growth and virulence. Microbiology (Reading) 146 (Pt 11):2755–2764. doi:10.1099/00221287-146-11-2755 11065354

[25] Ibe C, Munro CA. 2021. Fungal cell wall proteins and signaling pathways form a cytopropective network to combat stresses. J Fungi (Basel) 7:739. doi:10.3390/jof7090739 34575777 PMC8466366

[26] Román E, Correia I, Prieto D, Alonso R, Pla J. 2020. The HOG MAPK pathway in Candida albicans: more than an osmosensing pathway. Int Microbiol 23:23–29. doi:10.1007/s10123-019-00069-1 30875035

[27] Mottola A, Schwanfelder S, Morschhäuser J. 2020. Generation of viable Candida albicans mutants lacking the “essential” protein kinase Snf1 by inducible gene deletion. mSphere 5:e00805-20. doi:10.1128/mSphere.00805-20 32817381 PMC7440847

[28] Luther CH, Brandt P, Vylkova S, Dandekar T, Müller T, Dittrich M. 2023. Integrated analysis of SR-like protein kinases Sky1 and Sky2 links signaling networks with transcriptional regulation in Candida albicans. Front Cell Infect Microbiol 13:1108235. doi:10.3389/fcimb.2023.1108235 37082713 PMC10111165

[29] Huang G, Huang Q, Wei Y, Wang Y, Du H. 2019. Multiple roles and diverse regulation of the Ras/cAMP/protein kinase A pathway in Candida albicans. Mol Microbiol 111:6–16. doi:10.1111/mmi.14148 30299574

[30] Choi Y, Jeong E, Lee D-G, Jin J-H, So Y-S, Yu S-R, Lee K-J, Ha Y, Lin C-J, Chen Y-L, Park JB, Cho H-S, Averette AF, Heitman J, Lee K-H, Lee K, Bahn Y-S. 2022. Unraveling the pathobiological role of the fungal KEOPS complex in Cryptococcus neoformans. mBio 13:e0294422. doi:10.1128/mbio.02944-22 36377896 PMC9765431

[31] Du J, Dong Y, Zuo W, Deng Y, Zhu H, Yu Q, Li M. 2023. Mec1-Rad53 signaling regulates DNA damage-induced autophagy and pathogenicity in Candida albicans J Fungi (Basel) 9:1181. doi:10.3390/jof9121181 38132782 PMC10744610

[32] Coccetti P, Nicastro R, Tripodi F. 2018. Conventional and emerging roles of the energy sensor Snf1/AMPK in Saccharomyces cerevisiae. Microb Cell 5:482–494. doi:10.15698/mic2018.11.655 30483520 PMC6244292

[33] Woolford CA, Lagree K, Xu W, Aleynikov T, Adhikari H, Sanchez H, Cullen PJ, Lanni F, Andes DR, Mitchell AP. 2016. Bypass of Candida albicans filamentation/biofilm regulators though diminished expression of protein kinase Cak1. PLoS Genet 12:e1006487. doi:10.1371/journal.pgen.1006487 27935965 PMC5147786

[34] Wightman R, Bates S, Amornrrattanapan P, Sudbery P. 2004. In Candida albicans, the Nim1 kinases Gin4 and Hsl1 negatively regulate pseudohyphae formation and Gin4 also controls septin organization. J Cell Biol 164:581–591. doi:10.1083/jcb.200307176 14769857 PMC2171991

[35] Chiang LY, Sheppard DC, Bruno VM, Mitchell AP, Edwards JE, Filler SG. 2007. Candida albicans protein kinase CK2 governs virulence during oropharyngeal candidiasis. Cell Microbiol 9:233–245. doi:10.1111/j.1462-5822.2006.00784.x 16939537

[36] Alonso-Monge R, Navarro-García F, Molero G, Diez-Orejas R, Gustin M, Pla J, Sánchez M, Nombela C. 1999. Role of the mitogen-activated protein kinase Hog1 in morphogenesis and virulence of Candida albicans. J Bacteriol 181:3058–3068. doi:10.1128/JB.181.10.3058-3068.1999 10322006 PMC93760

[37] Navarro-García F, Alonso-Monge R, Rico H, Pla J, Sentandreu R, Nombela C. 1998. A role for the MAP kinase gene MKC1 in cell wall construction and morphological transitions in Candida albicans. Microbiology (Reading) 144 ( Pt 2):411–424. doi:10.1099/00221287-144-2-411 9493378

[38] Kumamoto CA. 2005. A contact-activated kinase signals Candida albicans invasive growth and biofilm formation. Proc Natl Acad Sci U S A 102:5576–5581. doi:10.1073/pnas.0407097102 15800048 PMC556227

[39] Saputo S, Chabrier-Rosello Y, Luca FC, Kumar A, Krysan DJ. 2012. The RAM network in pathogenic fungi. Eukaryot Cell 11:708–717. doi:10.1128/EC.00044-12 22544903 PMC3370468

[40] Braun BR, Kadosh D, Johnson AD. 2001. NRG1, a repressor of filamentous growth in C. albicans, is downregulated during filament induction. EMBO J 20:4753–4761. doi:10.1093/emboj/20.17.4753 11532939 PMC125265

[41] Murad AM, Leng P, Straffon M, Wishart J, Macaskill S, MacCallum D, Schnell N, Talibi D, Marechal D, Tekaia F, d’Enfert C, Gaillardin C, Odds FC, Brown AJ. 2001. NRG1 represses yeast-hypha morphogenesis and hypha-specific gene expression in Candida albicans. EMBO J 20:4742–4752. doi:10.1093/emboj/20.17.4742 11532938 PMC125592

[42] Kadosh D, Johnson AD. 2005. Induction of the Candida albicans filamentous growth program by relief of transcriptional repression: a genome-wide analysis. Mol Biol Cell 16:2903–2912. doi:10.1091/mbc.e05-01-0073 15814840 PMC1142434

[43] Saville SP, Lazzell AL, Monteagudo C, Lopez-Ribot JL. 2003. Engineered control of cell morphology in vivo reveals distinct roles for yeast and filamentous forms of Candida albicans during infection. Eukaryot Cell 2:1053–1060. doi:10.1128/EC.2.5.1053-1060.2003 14555488 PMC219382

[44] Lu Y, Su C, Unoje O, Liu H. 2014. Quorum sensing controls hyphal initiation in Candida albicans through Ubr1-mediated protein degradation. Proc Natl Acad Sci U S A 111:1975–1980. doi:10.1073/pnas.1318690111 24449897 PMC3918812

[45] Lu Y, Su C, Liu H. 2012. A GATA transcription factor recruits Hda1 in response to Tor1 signaling to establish a hyphal chromatin state in Candida albicans. PLoS Pathog 8:e1002663. doi:10.1371/journal.ppat.1002663 22536157 PMC3334898

[46] Wakade RS, Krysan DJ, Wellington M. 2022. Use of in vivo imaging to screen for morphogenesis phenotypes in Candida albicans mutant strains during active infection in a mammalian host model. J Vis Exp 188:e64258. doi:10.3791/64258 36314794

[47] Wakade RS, Wellington M, Krysan DJ. 2024. Temporal dynamics of Candida albicans morphogenesis and gene expression reveals distinctions between in vitro and in vivo filamentation. bioRxiv. doi:10.1101/2024.02.20.581211 PMC11036811 38501830

[48] Ramage G, Borghi E, Rodrigues CF, Kean R, Williams C, Lopez-Ribot J. 2023. Our current clinical understanding of Candida biofilms: where are we two decades on? APMIS 131:636–653. doi:10.1111/apm.13310 36932821

[49] Blankenship JR, Mitchell AP. 2006. How to build a biofilm: a fungal perspective. Curr Opin Microbiol 9:588–594. doi:10.1016/j.mib.2006.10.003 17055772

[50] Naseem S, Douglas LM, Konopka JB. 2019. Candida albicans rvs161Δ and rvs167Δ endocytosis mutants are defective in invasion into the oral cavity. mBio 10:e02503-19. doi:10.1128/mBio.02503-19 31719181 PMC6851284

[51] Uppuluri P, Acosta Zaldívar M, Anderson MZ, Dunn MJ, Berman J, Lopez Ribot JL, Köhler JR. 2018. Candida albicans dispersed cells are developmentally distinct from biofilm and planktonic cells. mBio 9:e01338-18. doi:10.1128/mBio.01338-18 30131358 PMC6106089

[52] Bonhomme J, Chauvel M, Goyard S, Roux P, Rossignol T, d’Enfert C. 2011. Contribution of glycolytic flux and hypoxia adaptation to efficient biofilm formation by Candida albicans. Mol Microbiol 80:995–1013. doi:10.1111/j.1365-2958.2011.07626.x 21414038

[53] Kowalski CH, Kerkaert JD, Liu KW, Bond MC, Hartmann R, Nadell CD, Stajich JE, Cramer RA. 2019. Fungal biofilm morphology impacts hypoxia fitness and disease progression. Nat Microbiol 4:2430–2441. doi:10.1038/s41564-019-0558-7 31548684 PMC7396965

[54] Gutiérrez-Escribano P, Zeidler U, Suárez MB, Bachellier-Bassi S, Clemente-Blanco A, Bonhomme J, Vázquez de Aldana CR, d’Enfert C, Correa-Bordes J. 2012. The NDR/LATS kinase Cbk1 controls the activity of the transcriptional regulator during biofilm formation in Candida albicans. PLoS Pathog 8:e1002683. doi:10.1371/journal.ppat.1002683 22589718 PMC3349750

[55] Wakade RS, Ristow LC, Stamnes MA, Kumar A, Krysan DJ. 2020. The Ndr/LATS kinase Cbk1 regulates a specific subset of Ace2 functions and suppresses the hypha-to yeast transition in Candida albicans. mBio 11:e01900-20. doi:10.1128/mBio.01900-20 32817109 PMC7439482

[56] Nobile CJ, Andes DR, Nett JE, Smith FJ, Yue F, Phan QT, Edwards JE, Filler SG, Mitchell AP. 2006. Critical role of Bcr1-dependent adhesins in C. albicans biofilm formation in vitro and in vivo. PLoS Pathog 2:e63. doi:10.1371/journal.ppat.0020063 16839200 PMC1487173

[57] Wakade RS, Huang M, Mitchell AP, Wellington M, Krysan DJ. 2021. Intravital imaging of Candida albicans identifies differential in vitro and in vivo filamentation phenotypes for transcription factor deletion mutants. mSphere 6:e0043621. doi:10.1128/mSphere.00436-21 34160243 PMC8265662

[58] Sharma A, Solis NV, Huang MY, Lanni F, Filler SG, Mitchell AP. 2023. Hgc1 independence of biofilm hyphae in Candida albicans. mBio 14:e0349822. doi:10.1128/mbio.03498-22 36779720 PMC10128054

[59] Mao Y, Solis NV, Sharma A, Cravener MV, Filler SG, Mitchell AP. 2022. Use of iron-responsive RBT5 promoter for regulated expression in Candida albicans. mSphere 7:e0030522. doi:10.1128/msphere.00305-22 35862800 PMC9429880

[60] Nett JE, Marchillo K, Andes DR. 2012. Modeling of fungal biofilms using a rat central vein catheter. Methods Mol Biol 845:547–556. doi:10.1007/978-1-61779-539-8_40 22328403 PMC3341608

[61] Desai JV, Bruno VM, Ganguly S, Stamper RJ, Mitchell KF, Solis N, Hill EM, Xu W, Filler SG, Andes DR, Fanning S, Lanni F, Mitchell AP. 2013. Regulatory role of glycerol in Candida albicans biofilm formation. mBio 4:e00637-12. doi:10.1128/mBio.00637-12 23572557 PMC3622937

[62] Roskoski R. 2024. Properties of FDA-approved small molecule protein kinase inhibitors: a 2024 update. Pharmacol Res 200:107059. doi:10.1016/j.phrs.2024.107059 38216005

[63] González-Rubio G, Sellers-Moya Á, Martín H, Molina M. 2021. A walk-through MAPK structure and functionality with the 30-year-old MAPK Slt2. Int Microbiol 24:531–543. doi:10.1007/s10123-021-00183-z 33993419

[64] Ramírez-Zavala B, Krüger I, Wollner A, Schwanfelder S, Morschhäuser J. 2023. The Ypk1 protein kinase pathway is rewired and not essential for viability in Candida albicans. PLoS Genet 19:e1010890. doi:10.1371/journal.pgen.1010890 37561787 PMC10443862

[65] Lee Y, Liston SD, Lee D, Robbins N, Cowen LE. 2022. Functional analysis of the Candida albicans kinome reveals Hrr25 as a regulator antifungal susceptibility. iScience 25:104432. doi:10.1016/j.isci.2022.104432 35663022 PMC9160768

[66] Mao Y, Solis NV, Filler SG, Mitchell AP. 2023. Functional dichotomy for a hyphal repressor in Candida albicans. mBio 14:e0013423. doi:10.1128/mbio.00134-23 36883818 PMC10127614

[67] Wakade RS, Wellington M, Krysan DJ. 2024. The role of the C. albicans transcriptional repressor NRG1 during filamentation and disseminated candidiasis is strain-dependent. bioRxiv. doi:10.1101/2023.12.15.571891 PMC10964420 38376205

[68] Wang Y. 2016. How much does a single protein kinase do in the regulation of hyphal development in Candida albicans? J Microbiol 54:170–177. doi:10.1007/s12275-016-5550-9 26920877

[69] Gutiérrez-Escribano P, González-Novo A, Suárez MB, Li C-R, Wang Y, de Aldana CRV, Correa-Bordes J. 2011. CDK-dependent phosphorylation of Mob2 is essential for hyphal development in Candida albicans. Mol Biol Cell 22:2458–2469. doi:10.1091/mbc.E11-03-0205 21593210 PMC3135472

[70] Willger SD, Liu Z, Olarte RA, Adamo ME, Stajich JE, Myers LC, Kettenbach AN, Hogan DA. 2015. Analysis of the Candida albicans phophoproteome. Eukaryot Cell 14:474–485. doi:10.1128/EC.00011-15 25750214 PMC4421004

[71] Cao C, Wu M, Bing J, Tao L, Ding X, Liu X, Huang G. 2017. Global regulatory roles of the cAMP/PKA pathway revealed by phenotypic, transcriptomic, and phophoproteomic analyses in a null mutant of the PKA catalytic subunit in Candida albicans. Mol Microbiol 105:46–64. doi:10.1111/mmi.13681 28370450

[72] Min K, Jannace TF, Si H, Veeramah KR, Haley JD, Konopka JB. 2021. Integrative multi-omics profiling reveals cAMP-independent mechanisms regulating hyphal morphogenesis. PLoS Pathog 17:e1009861. doi:10.1371/journal.ppat.1009861 34398936 PMC8389844

[73] Lee HJ, Kim JM, Kang WK, Yang H, Kim JY. 2015. The NDR kinase Cbk1 downregulates the transcriptional repressor Nrg1 through the mRNA-binding protein Ssd1 in Candida albicans. Eukaryot Cell 14:671–683. doi:10.1128/EC.00016-15 26002720 PMC4486675

[74] Huang MY, Cravener MC, Mitchell AP. 2021. Targeted genetic changes in Candida albicans using transient CRISPR-Cas9 expression. Curr Protoc 1:e19. doi:10.1002/cpz1.19 33491919 PMC7842826

[75] Wilson RB, Davis D, Mitchell AP. 1999. Rapid hypothesis testing with Candida albicans through gene disruption with short homology regions. J Bacteriol 181:1868–1874. doi:10.1128/JB.181.6.1868-1874.1999 10074081 PMC93587

[76] Glazier VE, Murante T, Murante D, Koselny K, Liu Y, Kim D, Koo H, Krysan DJ. 2017. Genetic analysis of the Candida albicans biofilm transcription factor network using simple and complex haploinsufficiency. PLoS Genet 13:e1006948. doi:10.1371/journal.pgen.1006948 28793308 PMC5565191

[77] Chen T, Wagner AS, Tams RN, Eyer JE, Kauffman SJ, Gann ER, Fernandez EJ, Reynolds TB. 2019. Lrg1 regulates β (1,3)-glucan masking in Candida albicans through the Cek1 MAP kinase pathway. mBio 10:e01767-19. doi:10.1128/mBio.01767-19 31530671 PMC6751057

[78] Saputo S, Kumar A, Krysan DJ. 2014. Efg1 directly regulates ACE2 expression to mediate cross talk between the cAMP/PKA and RAM pathways during Candida albicans morphogenesis. Eukaryot Cell 13:1169–1180. doi:10.1128/EC.00148-14 25001410 PMC4187626

