# Supplementary figures and images for: Systematic analysis of the Candida albicans kinome reveals environmentally contingent protein kinase-mediated regulation of filamentation and biofilm formation in vitro and in vivo
Source: mBio. 2024 Jul 1;15(8):e01249-24. doi: 10.1128/mbio.01249-24 (PMC11323567; doi:10.1128/mbio.01249-24)

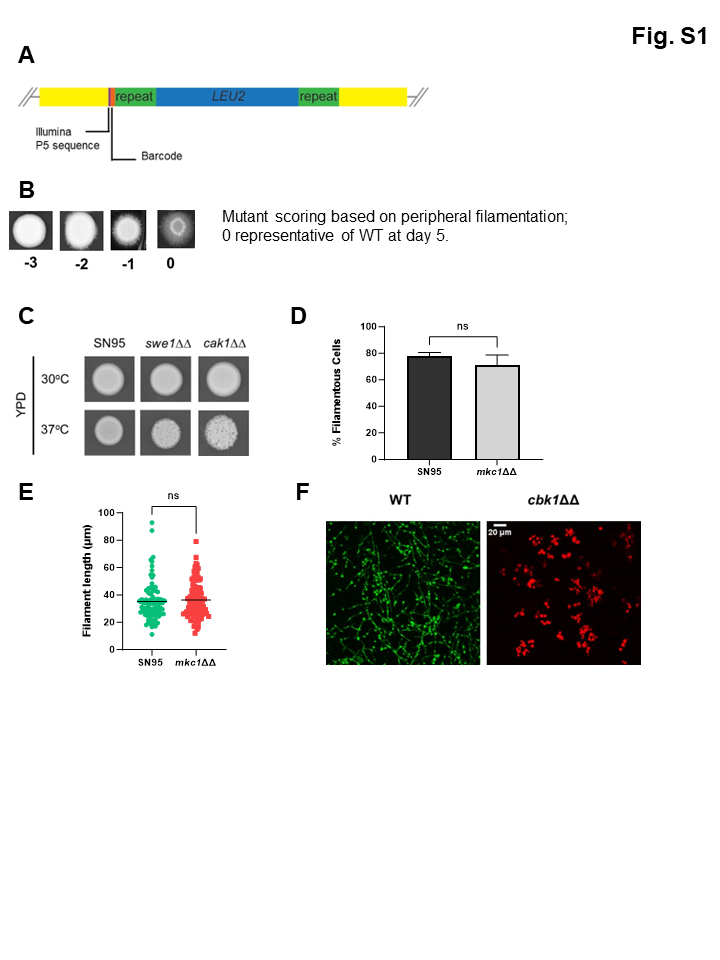

Supplement: Fig. S1 — Filamentation phenotypes. [file mbio.01249-24-s0001.tif]

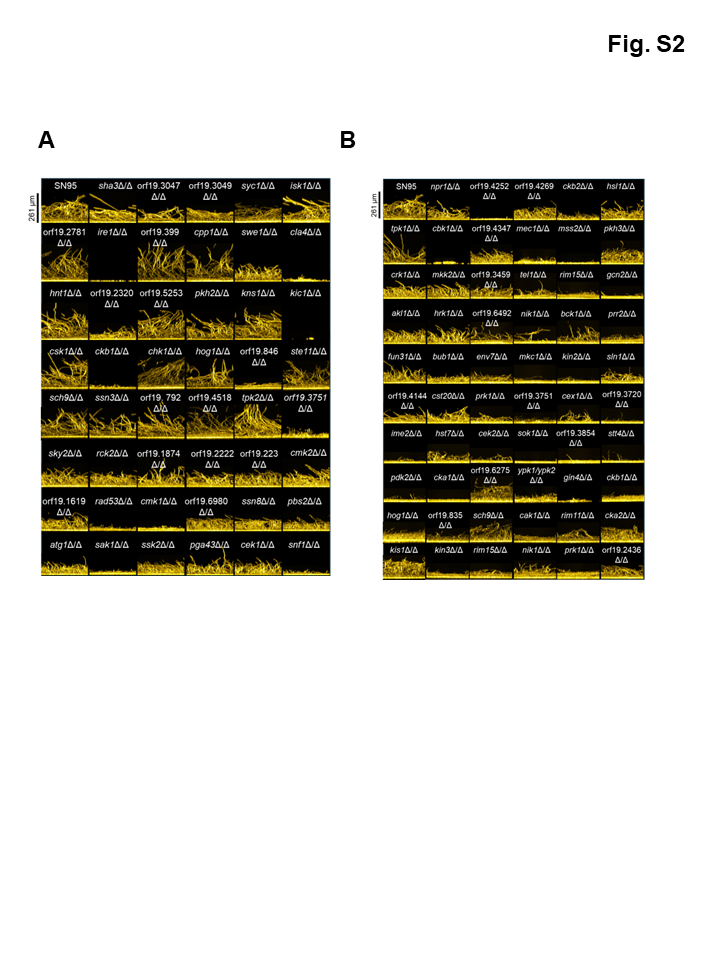

Supplement: Fig. S2 — Biofilms for all 99 PK mutants. [file mbio.01249-24-s0002.tif]

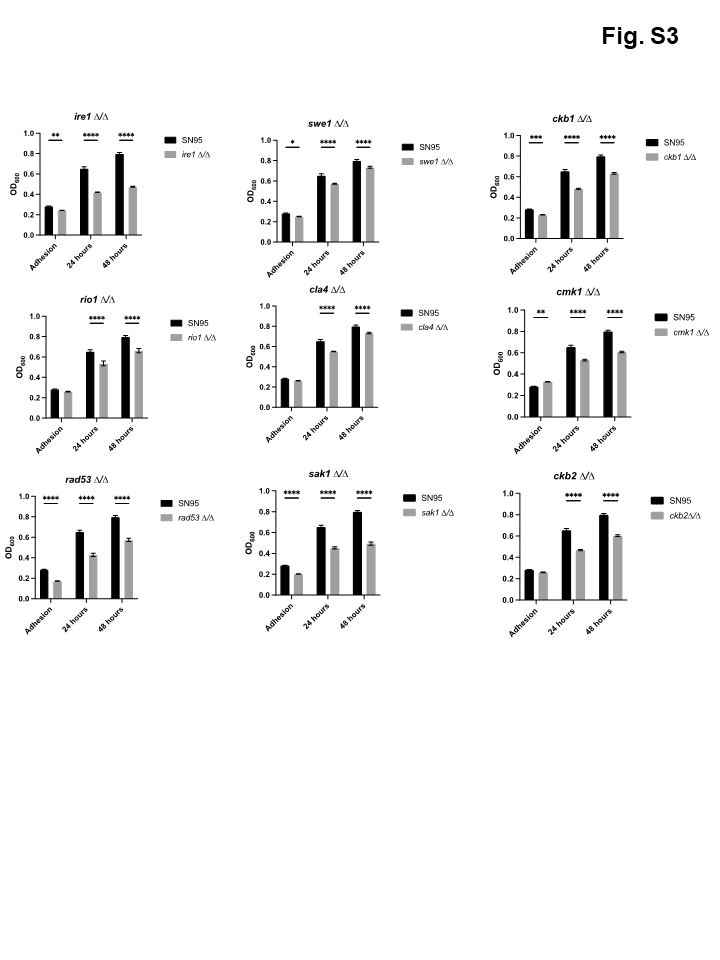

Supplement: Fig. S3 — Biofilm density data for selected mutants. [file mbio.01249-24-s0003.tif]

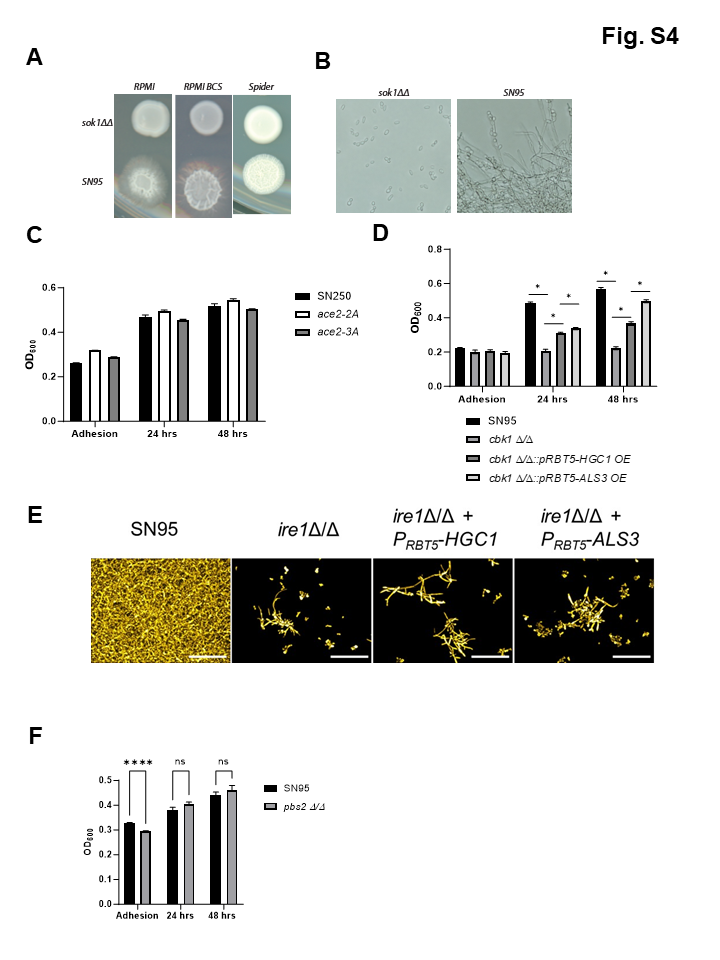

Supplement: Fig. S4 — Biofilm phenotypes of RAM pathway and SOK1 mutants. [file mbio.01249-24-s0004.tif]
