# Supplementary material for: Systematic analysis of the Candida albicans kinome reveals environmentally contingent protein kinase-mediated regulation of filamentation and biofilm formation in vitro and in vivo
Source: mBio. 2024 Jul 1;15(8):e01249-24. doi: 10.1128/mbio.01249-24 (PMC11323567; doi:10.1128/mbio.01249-24)
Supplement: Legends — for supplemental material. [file mbio.01249-24-s0005.docx]

**Figure S1.** **A**. Schematic of deletion construct sequence. **B**. Examples of scoring scheme for filamentation. **C**. Example phenotypes of two protein kinase mutants, *swe1*∆∆ and *cak1*∆∆, with increased filamentation on YPD at 37^o^C. Representative images of two independent replicates. **D**. The percentage of filamentous cells for SN95 and the *mkc1*∆∆ mutant 24hr after infection. The bars are median of data from three independent experiments. NS (p >0.05) not significant by Student’s t test. **E**. Length of the filaments from three independent experiments. NS not significant (p>0.05) by Mann Whitney U test. **F.** In vivo images of WT and *cbk1*∆∆ mutant representative of data shown in Fig. 5F.

**Figure S2. A&B**. Representative images of biofilms for all 99 protein kinase mutants in RPMI+10% bovine calf serum at 24hr.

**Figure S3**. Initial adhesion and 24/49hr biofilm density for selected mutants from imaging screen. The indicated strains were incubated in microtiter plates in RPMI medium at 37^o^C. For adhesion, the plates were washed with PBS at 90 minutes and the OD_600_ determined. Mature biofilms were measured at 24hr and 48hr. Error bars indicate mean of 4-5 replicates with error bars standard deviation. **** indicates statistical significance (p <0.05) by two-way ANOVA with correction for multiple comparisons.

**Figure S4.** **A**. Filamentation phenotypes on solid RPMI, RPMI+10% BCS, and Spider medium at 37^o^C for the *sok1*∆∆ mutant. **B**. SN95 and *sok1*∆∆ were inoculated into a 6-well dish RPMI+10%BCS medium containing a microscope cover slip at 37^o^C. The coverslip was removed and washed with PBS to dislodge unadhered cells. The strains were then imaged by light microscopy. **C.** Biofilm density of the indicated strains after adhesion and 24 or 48hr maturation. The bars indicate mean of 4-5 replicates with error bars indicating standard deviation. There were no statistically significant differences between the *ace2* mutants and the SN250 reference strain. **D**. Biofilm density assay for *cbk1*∆∆ mutant and the corresponding P*_RBT5_*-*ALS3*/*HGC1* derivatives. * indicates p <0.05 for 2-way ANOVA with correction for multiple comparisons. **E**. Apical images of SN95 and *ire1*∆∆ mutants along with the corresponding P*_RBT5_*-*ALS3*/*HGC1* derivatives after 24hr incubation in RPMI+10%BCS. **F**. The effect of 2% glycerol on the biofilm density of the *pbs2*∆∆ mutant. NS indicates not significant by two-way ANOVA with correction for multiple comparisons.

**Table S1**. Master list of protein kinase mutants, barcodes relating to their identity, oligonucleotides used in their construction, and oligonucleotides used to confirm correct genotype.

**Table S2.** List of protein kinases for which only heterozygotes were obtained.

**Table S3**. Competitive fitness data for protein kinases mutants at 30^o^C and under 10 different growth conditions. Competitive fitness data for three replicates, average, and statistical significance.

**Table S4**. Summary of protein kinases with reduced fitness in each condition.

**Table S5**. Summary of filamentation and biofilm phenotypes for protein kinase mutants.
